# Supplementary material for: A Powerful Procedure for Pathway-Based Meta-analysis Using Summary Statistics Identifies 43 Pathways Associated with Type II Diabetes in European Populations
Source: PLoS Genet. 2016 Jun 30;12(6):e1006122. doi: 10.1371/journal.pgen.1006122 (PMC4928884; doi:10.1371/journal.pgen.1006122)
Supplement: S1 Table — (DOCX) [file pgen.1006122.s001.docx]

S1 Table. Power comparison between sARTP and aSPUsPath under the scenrio where each outcome-associated gene contains one functional SNP.

|  |  | Pathway with 20 genes | | |  | Pathway with 50 genes | | |  | Pathway with 80 genes | | |
| --- | --- | --- | --- | --- | --- | --- | --- | --- | --- | --- | --- | --- |
| $r\%$ |  | $\gamma^{*}$ | sARTP | aSPUsPath |  | $\gamma^{*}$ | sARTP | aSPUsPath |  | $\gamma^{*}$ | sARTP | aSPUsPath |
| 5 |  | 0.32 | 0.581 | 0.493 |  | 0.28 | 0.717 | 0.638 |  | 0.26 | 0.617 | 0.572 |
| 5 |  | ±0.32 | 0.581 | 0.493 |  | ±0.28 | 0.708 | 0.605 |  | ±0.26 | 0.594 | 0.517 |
| 10 |  | 0.28 | 0.694 | 0.605 |  | 0.24 | 0.702 | 0.607 |  | 0.22 | 0.672 | 0.574 |
| 10 |  | ±0.28 | 0.626 | 0.551 |  | ±0.24 | 0.680 | 0.581 |  | ±0.22 | 0.630 | 0.532 |
| 20 |  | 0.23 | 0.666 | 0.579 |  | 0.20 | 0.675 | 0.622 |  | 0.18 | 0.634 | 0.549 |
| 20 |  | ±0.23 | 0.620 | 0.524 |  | ±0.20 | 0.675 | 0.579 |  | ±0.18 | 0.608 | 0.509 |
| 30 |  | 0.20 | 0.609 | 0.550 |  | 0.18 | 0.709 | 0.665 |  | 0.16 | 0.618 | 0.582 |
| 30 |  | ±0.20 | 0.572 | 0.494 |  | ±0.18 | 0.663 | 0.574 |  | ±0.16 | 0.576 | 0.509 |

For each simulation setting, the empirical powers are computed from 1,000 simulated datasets at the level of 0.05. Each dataset consists of 1,000 cases and 1,000 controls.

The proportion of functional gene(s) in a pathway;

Log odds ratio of functional SNPs in the underlying risk models. These values are chosen to ensure considered tests to have appropriate powers;

Log odds ratios of functional SNPs are either positive or negative with equal probability.
