# Supplementary material for: A Powerful Procedure for Pathway-Based Meta-analysis Using Summary Statistics Identifies 43 Pathways Associated with Type II Diabetes in European Populations
Source: PLoS Genet. 2016 Jun 30;12(6):e1006122. doi: 10.1371/journal.pgen.1006122 (PMC4928884; doi:10.1371/journal.pgen.1006122)
Supplement: S3 Table — (DOCX) [file pgen.1006122.s003.docx]

S3 Table: Summary of top 50 genes with smallest gene-level p-values from the gene-level meta-analysis based on the DIAGRAM and GERA studies.

| Gene | Chromosome | META | DIAGRAM | GERA |
| --- | --- | --- | --- | --- |
| ATP5G1 | 17 | 2.90E-07 | 2.08E-03 | 2.91E-05 |
| SNF8 | 17 | 3.40E-07 | 1.00E-03 | 4.87E-05 |
| UBE2Z | 17 | 3.60E-07 | 1.59E-03 | 5.13E-05 |
| GIP | 17 | 6.40E-07 | 1.02E-03 | 2.40E-04 |
| SLC2A2 | 3 | 8.97E-06 | 9.97E-03 | 2.95E-04 |
| MYBL2 | 20 | 9.84E-06 | 4.41E-04 | 7.07E-03 |
| IFT52 | 20 | 2.00E-05 | 2.71E-04 | 3.26E-03 |
| SREBF1 | 17 | 3.09E-05 | 1.45E-02 | 1.62E-03 |
| SMEK1 | 14 | 4.65E-05 | 9.53E-05 | 7.41E-02 |
| TACO1 | 17 | 4.84E-05 | 1.86E-03 | 4.77E-03 |
| SOCS2 | 12 | 4.94E-05 | 7.76E-02 | 2.88E-04 |
| IL20RA | 6 | 5.29E-05 | 5.59E-03 | 1.61E-02 |
| PLEKHA1 | 10 | 6.94E-05 | 7.23E-05 | 3.82E-02 |
| YPEL2 | 17 | 7.41E-05 | 3.60E-03 | 1.73E-02 |
| RAB1A | 2 | 8.05E-05 | 3.97E-03 | 1.89E-02 |
| CDKN2C | 1 | 8.11E-05 | 1.20E-02 | 3.61E-03 |
| CENPW | 6 | 9.15E-05 | 1.96E-03 | 2.70E-02 |
| RAI1 | 17 | 9.20E-05 | 8.82E-03 | 6.64E-03 |
| C11orf34 | 11 | 1.12E-04 | 1.14E-02 | 4.59E-03 |
| KIAA0754 | 1 | 1.16E-04 | 8.05E-02 | 7.38E-04 |
| ACSL1 | 4 | 1.19E-04 | 3.87E-03 | 4.52E-02 |
| UBAP2 | 9 | 1.38E-04 | 9.94E-03 | 2.40E-02 |
| MACF1 | 1 | 1.51E-04 | 1.14E-01 | 2.09E-04 |
| MAP3K3 | 17 | 1.51E-04 | 4.88E-03 | 2.01E-02 |
| GTSF1L | 20 | 1.59E-04 | 4.67E-03 | 1.91E-02 |
| MIR17HG | 13 | 1.66E-04 | 1.39E-02 | 5.80E-03 |
| SGK2 | 20 | 1.68E-04 | 5.05E-04 | 5.56E-02 |
| CEP68 | 2 | 1.75E-04 | 9.38E-03 | 2.00E-02 |
| BLOC1S2 | 10 | 1.84E-04 | 5.75E-04 | 1.07E-01 |
| PABPC4 | 1 | 1.97E-04 | 1.33E-01 | 9.85E-04 |
| PPIEL | 1 | 2.10E-04 | 1.29E-01 | 1.03E-03 |
| CALCOCO2 | 17 | 2.29E-04 | 5.50E-02 | 1.41E-03 |
| YTHDC2 | 5 | 2.37E-04 | 6.21E-03 | 4.33E-02 |
| ARNTL | 11 | 2.54E-04 | 4.30E-02 | 2.03E-02 |
| DCAF7 | 17 | 2.73E-04 | 6.11E-03 | 1.61E-02 |
| PTS | 11 | 2.89E-04 | 7.46E-03 | 2.33E-03 |
| PDE3B | 11 | 3.06E-04 | 4.44E-01 | 2.99E-04 |
| ZNF276 | 16 | 3.08E-04 | 9.09E-02 | 7.26E-05 |
| C16orf7 | 16 | 3.60E-04 | 1.11E-01 | 8.42E-05 |
| FANCA | 16 | 4.31E-04 | 1.03E-01 | 9.35E-05 |
| CWF19L1 | 10 | 4.43E-04 | 7.50E-04 | 8.81E-02 |
| GPR151 | 5 | 4.68E-04 | 3.22E-02 | 1.04E-03 |
| KCNH6 | 17 | 4.82E-04 | 1.41E-02 | 2.55E-02 |
| EIF5A2 | 3 | 4.93E-04 | 6.43E-02 | 1.47E-03 |
| FEN1 | 11 | 5.11E-04 | 1.06E-02 | 2.27E-02 |
| CPPED1 | 16 | 5.21E-04 | 5.05E-03 | 7.08E-02 |
| ZNF664 | 12 | 5.35E-04 | 2.61E-02 | 2.29E-02 |
| C11orf10 | 11 | 5.46E-04 | 1.06E-02 | 2.47E-02 |
| LIMD2 | 17 | 5.50E-04 | 1.52E-02 | 2.85E-02 |
| CHUK | 10 | 5.59E-04 | 8.33E-04 | 1.23E-01 |

The top 50 genes are chosen from 15,946 unique genes included in 4,713 candidate pathways for having smallest gene-level p-values based on the gene-level meta-analysis.

P-values from the gene-level meta-analysis combining summary statistics from the DIAGRAM and GERA studies;

P-values from the gene-level analysis using summary statistics from the DIAGRAM study;

P-values from the gene-level analysis using summary statistics from the GERA study.
