# Supplementary material for: A Powerful Procedure for Pathway-Based Meta-analysis Using Summary Statistics Identifies 43 Pathways Associated with Type II Diabetes in European Populations
Source: PLoS Genet. 2016 Jun 30;12(6):e1006122. doi: 10.1371/journal.pgen.1006122 (PMC4928884; doi:10.1371/journal.pgen.1006122)
Supplement: S4 Table — (DOCX) [file pgen.1006122.s004.docx]

S4 Table. Effect of SNP rs1058018 on type 2 diabetes.

| Study | OR | 95% CI | P | EAF |
| --- | --- | --- | --- | --- |
| DIAGRAM | 0.93 | [0.90, 0.97] | 4.40E-4 | n/a |
| GERA | 0.91 | [0.88, 0.95] | 4.60E-6 | 0.43 |
| DIAGRAM+GERA | 0.92 | [0.90, 0.95] | 3.06E-8 | n/a |
| AGEN | 0.97 | [0.91, 1.03] | 0.29 | n/a |

odds ratio, with the allele ‘T’ as the reference allele, and ‘C’ as the effect allele;

95% confidence interval, adjusted for the genomic control inflation factor;

P-value, adjusted for the genomic control inflation factor;

effect allele frequency, unavailable in the DIAGRAM and AGEN data, but reported to be 0.40 in EUR, and 0.25 in EAS accordingly to the 1000 Genomes Project (phase 3).
