# Supplementary material for: A Powerful Procedure for Pathway-Based Meta-analysis Using Summary Statistics Identifies 43 Pathways Associated with Type II Diabetes in European Populations
Source: PLoS Genet. 2016 Jun 30;12(6):e1006122. doi: 10.1371/journal.pgen.1006122 (PMC4928884; doi:10.1371/journal.pgen.1006122)
Supplement: S5 Table — (DOCX) [file pgen.1006122.s005.docx]

S5 Table. The genomic control inflation factors, Spearman’s rank correlation coefficients between the pathway size and its p-value based on results obtained by applying sARTP to 20 simulated GWAS under the null.

| Experiment | $\lambda^{a}$ | $r_{s}^{b}$ | $r_{s}^{c}$ |
| --- | --- | --- | --- |
| 1 | 0.75 | 0.072 | 0.047 |
| 2 | 0.70 | 0.081 | 0.044 |
| 3 | 0.90 | 0.033 | 0.033 |
| 4 | 0.65 | 0.020 | 0.012 |
| 5 | 0.51 | 0.10 | 0.11 |
| 6 | 1.03 | -0.0038 | 0.021 |
| 7 | 0.94 | 0.017 | 0.0034 |
| 8 | 1.08 | -0.0068 | 0.0037 |
| 9 | 1.04 | -0.084 | -0.090 |
| 10 | 0.79 | 0.040 | 0.033 |
| 11 | 1.59 | -0.11 | -0.12 |
| 12 | 1.16 | -0.043 | -0.026 |
| 13 | 1.07 | -0.015 | 0.0064 |
| 14 | 1.19 | -0.087 | -0.087 |
| 15 | 0.96 | -0.0043 | 0.0071 |
| 16 | 1.40 | -0.052 | -0.042 |
| 17 | 1.23 | -0.039 | -0.052 |
| 18 | 0.96 | 0.039 | 0.039 |
| 19 | 0.94 | -0.030 | -0.022 |
| 20 | 0.82 | 0.052 | 0.046 |

genomic control inflation factor;

Spearman’s rank correlation coefficients between the number of unique SNPs in a pathway and the pathway p-value;

Spearman’s rank correlation coefficients between the number of genes in a pathway and the pathway p-value.
