# Supplementary material for: A Powerful Procedure for Pathway-Based Meta-analysis Using Summary Statistics Identifies 43 Pathways Associated with Type II Diabetes in European Populations
Source: PLoS Genet. 2016 Jun 30;12(6):e1006122. doi: 10.1371/journal.pgen.1006122 (PMC4928884; doi:10.1371/journal.pgen.1006122)
Supplement: S1 Text — (DOCX) [file pgen.1006122.s008.docx]

**S1 Text. Recovering score statistics and their variance-covariance matrix using summary results from the fixed effects model**

Here we derive the approximated score statistic $S$ and its variance-covariance matrix $V$ using summary statistics from the fixed effect model. Based on (4) in the main text, it is straightforward to see that $S_{t}\approx\tau_{t}^{-2}\hat{\beta}_{t}$. Note that $V_{ts}$ in equation (5) depends on $\tau_{t}^{(l)}$ estimated from individual studies, which cannot be derived from $\tau_{t}$. However, assume that $\sqrt{n_{t}^{(l)}}\tau_{t}^{(l)}$ can be approximated as an unknown but common constant value $\nu_{t}$ across all studies, and if $\bar{y}^{(l)}\left(1-\bar{y}^{(l)}\right)\approx\bar{y}\left(1-\bar{y}\right)$, we have $\nu_{t}\approx\sqrt{n_{t}}\tau_{t}$, and $V_{ts}\approx\frac{n_{ts}}{\sqrt{n_{t}n_{s}}}\frac{\rho_{ts}}{\tau_{t}\tau_{s}}$. The similar argument has been used in [1] to demonstrate that the meta-analysis is as efficient as the pooled analysis under those conditions.

1. Lin DY, Zeng D. On the relative efficiency of using summary statistics versus individual-level data in meta-analysis. Biometrika. 2010;97(2):321-32.
