# Supplementary material for: A Powerful Procedure for Pathway-Based Meta-analysis Using Summary Statistics Identifies 43 Pathways Associated with Type II Diabetes in European Populations
Source: PLoS Genet. 2016 Jun 30;12(6):e1006122. doi: 10.1371/journal.pgen.1006122 (PMC4928884; doi:10.1371/journal.pgen.1006122)
Supplement: S2 Text — (DOCX) [file pgen.1006122.s009.docx]

**S2 Text. Further evaluation of sARTP under the null**

We conducted additional experiments to evaluate the empirical size of sARTP. Based on the GERA T2D GWAS, we created 20 GWAS data under the null by randomly permuting the outcome, while keeping individual genotypes unchanged. On each null data, we excluded 274 pathways with over 10,000 SNPs for the sake of reducing computational burden, and conducted a pathway-based meta-analysis with sARTP on the remaining 4,439 pathways defined in MSigDB v5.0. The Q-Q plots of the pathway p-values of these 20 experiments are shown in S50 Fig. Since there are extensive overlaps between pathways, their pathway p-values in each experiment are correlated. As a result, the Q-Q plot has a large variation around the diagonal line. But on average, there is no apparent genomic control inflation across 20 experiments. Based on those 20 experiments, S5 Table shows the genomic control inflation factors, Spearman’s rank correlation coefficient between the pathway size (in terms of the number of unique SNPs, or genes in a pathway) and its pathway p-value. By inspecting those correlation coefficients, we did not see any evidence suggesting that the association significance level of a pathway is influenced by its size under the null.
