# Supplementary figures and images for: A Powerful Procedure for Pathway-Based Meta-analysis Using Summary Statistics Identifies 43 Pathways Associated with Type II Diabetes in European Populations
Source: PLoS Genet. 2016 Jun 30;12(6):e1006122. doi: 10.1371/journal.pgen.1006122 (PMC4928884; doi:10.1371/journal.pgen.1006122)

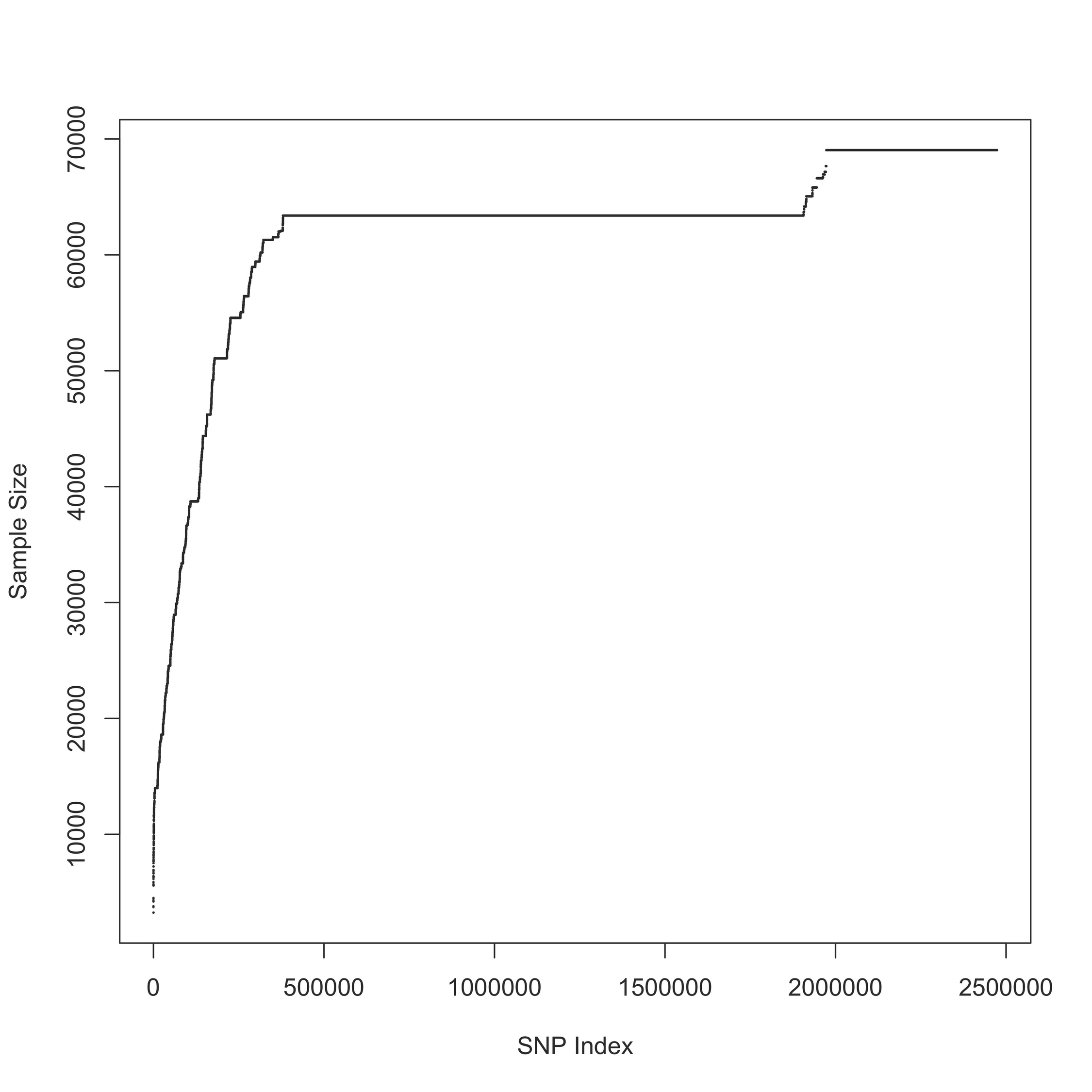

Supplement: S1 Fig — The SNP index (x-axis) is sorted according to its meta-analysis sample size. There are 2.5 million autosomal SNPs genotyped or imputed in at least one of the twelve participating GWAS in the DIAGRAM meta-analysis. 19.0% of those SNPs have a sample size of 69,033 (12,171 cases and 56,862 controls), which is total sample size of all twelve participating GWAS combined. Another 57.9% of those SNPs have a sample size of 63,390 (9,580 cases and 53,810). (TIF) [file pgen.1006122.s010.tif]

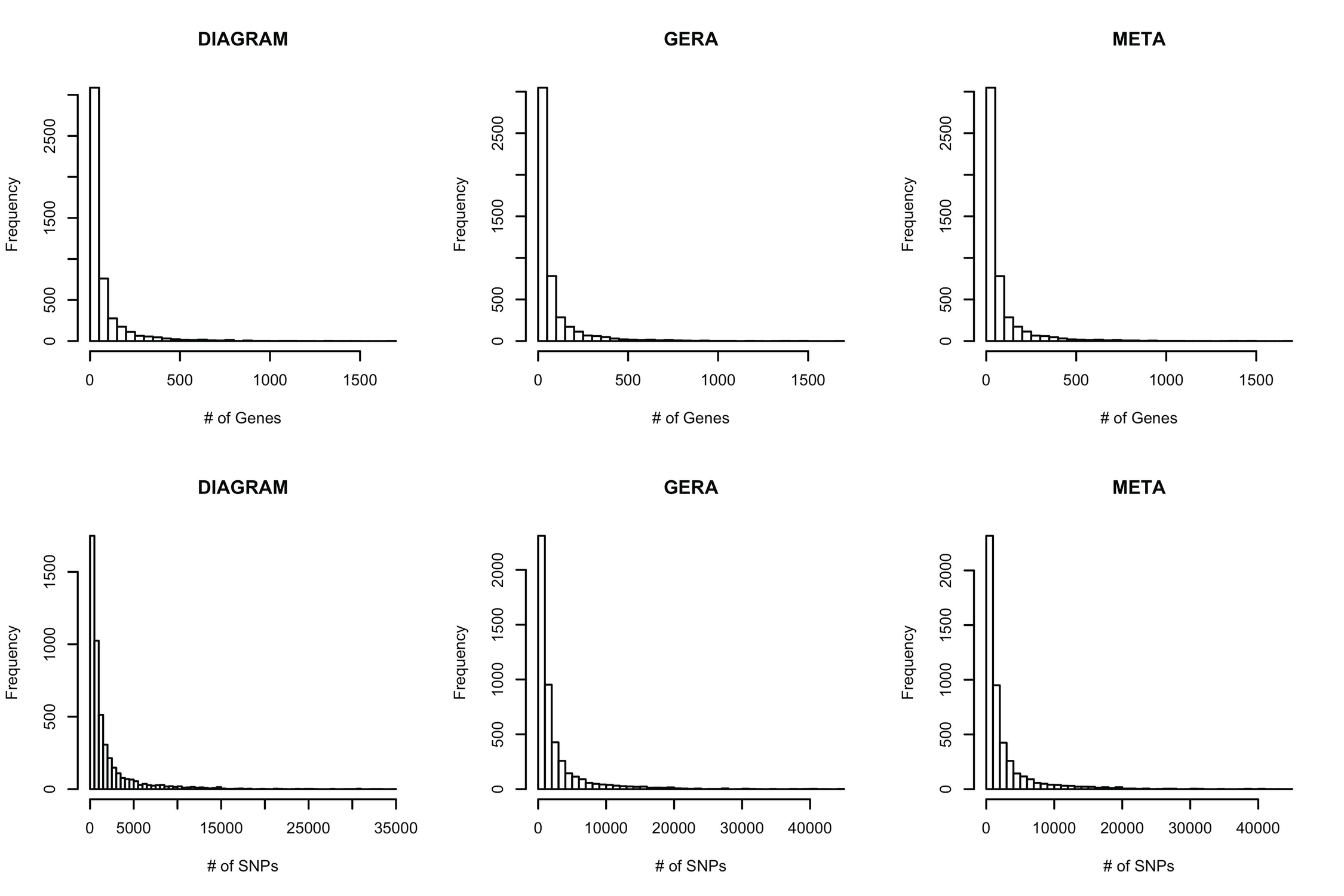

Supplement: S2 Fig — (TIF) [file pgen.1006122.s011.tif]

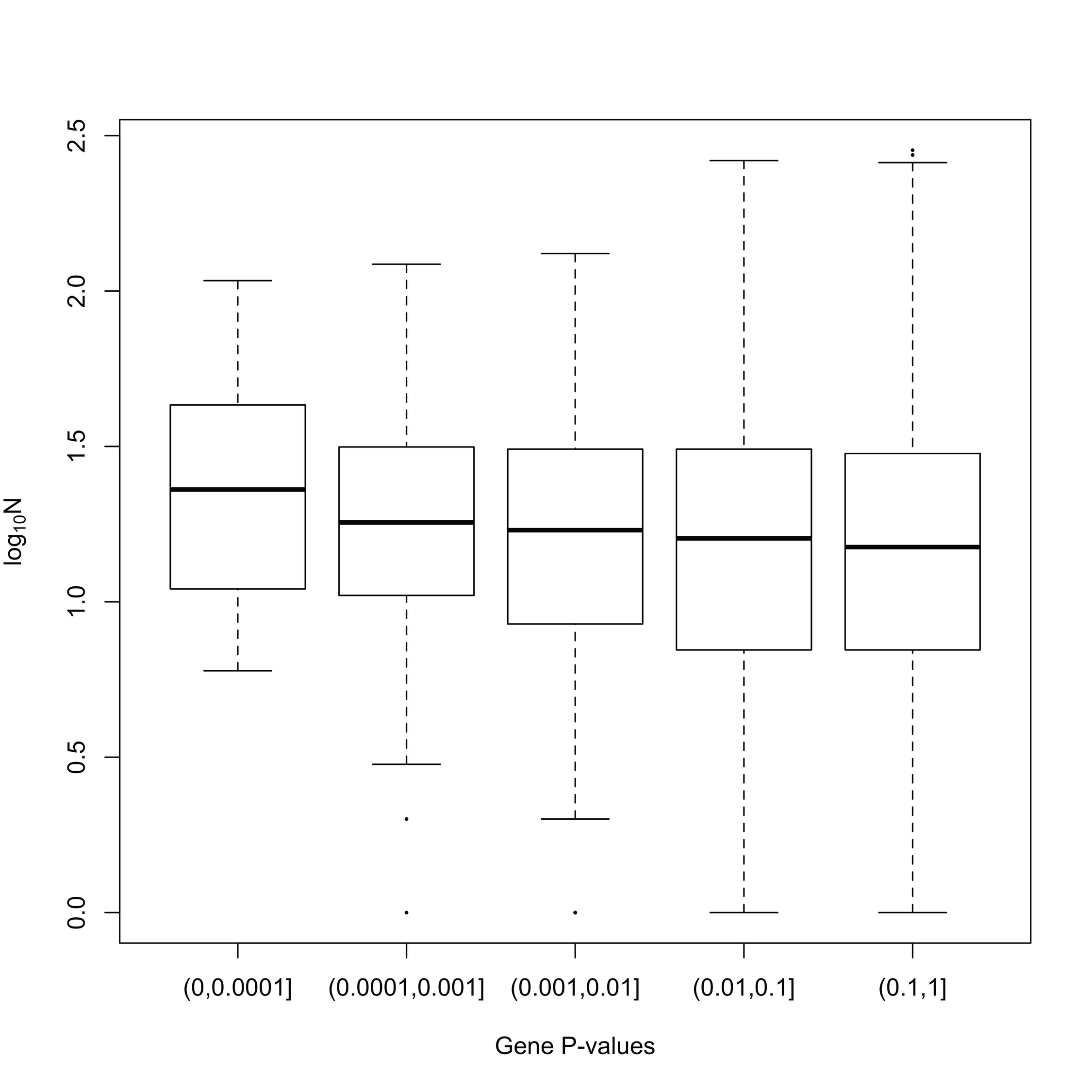

Supplement: S3 Fig — Genes are stratified into five groups according to their p-values from the gene-level meta-analysis on the summary data from the DIAGRAM and GERA studies. The boxplot summarizes the number of pathways containing a gene within a given group. (TIF) [file pgen.1006122.s012.tif]

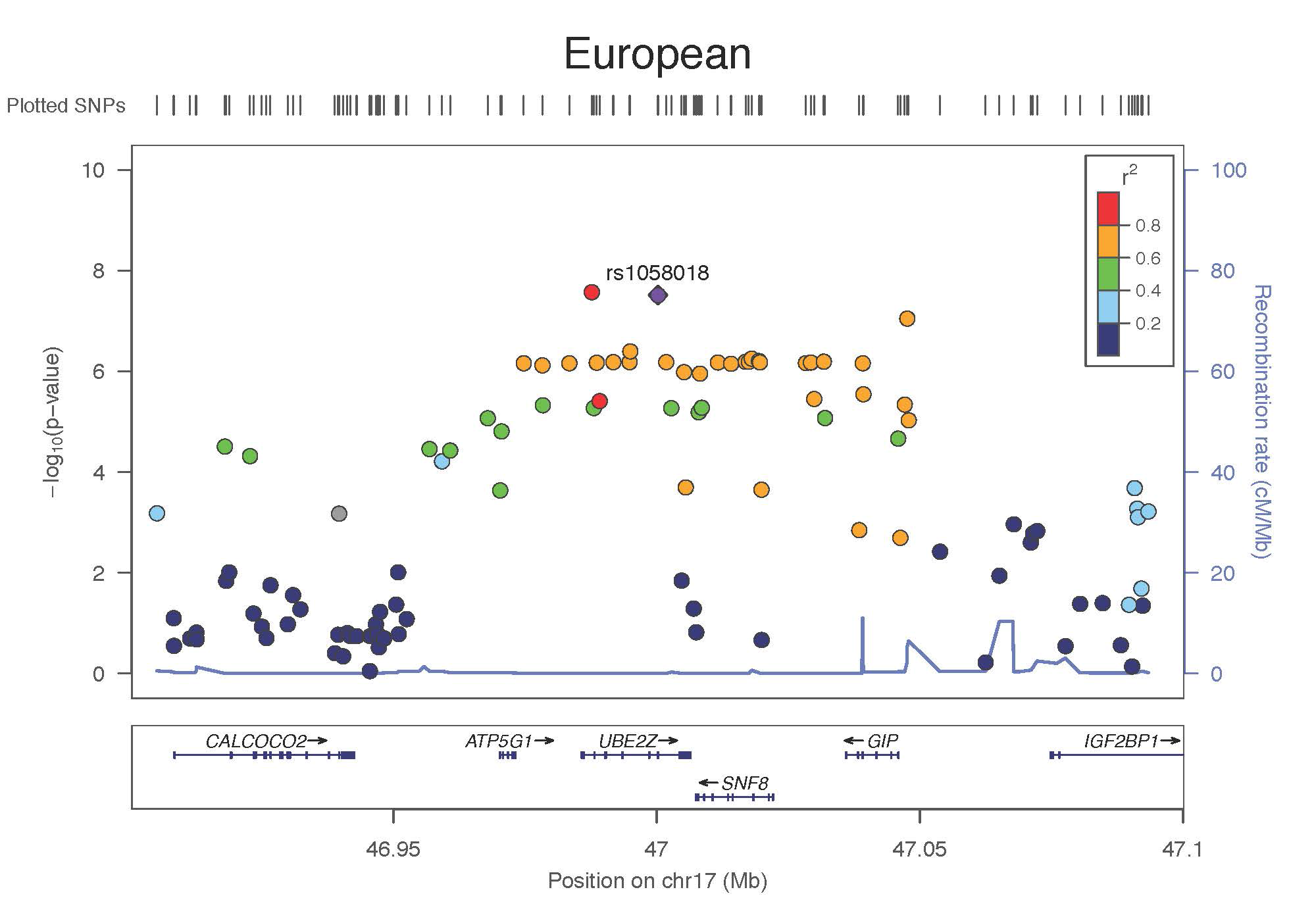

Supplement: S4 Fig — The SNP p-values were computed based on combined data of DIAGRAM and GERA studies after two rounds of genomic control inflation factor adjustment. (TIF) [file pgen.1006122.s013.tif]

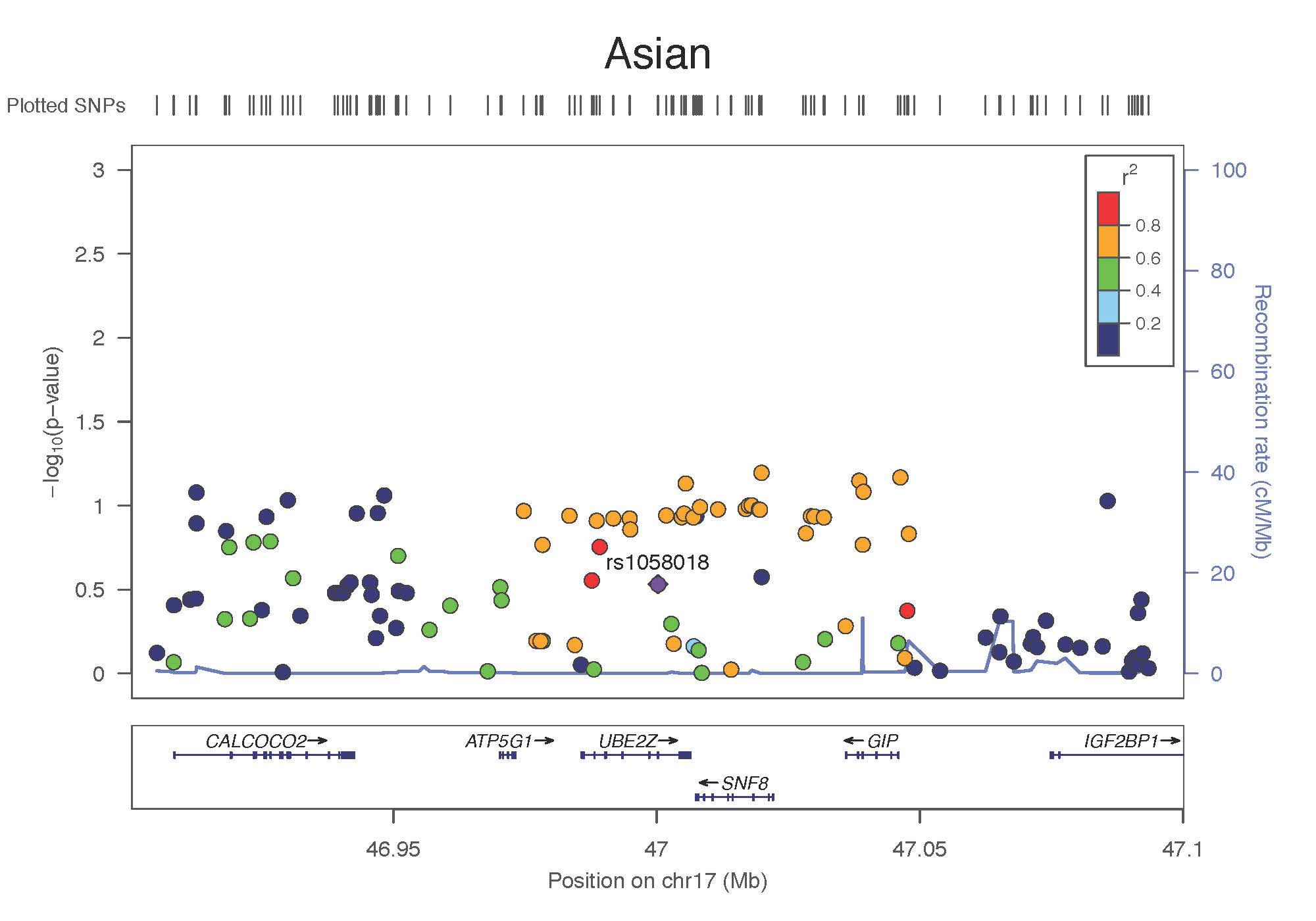

Supplement: S5 Fig — The SNP p-values were computed based on data of AGEN-T2D study after the adjustment of genomic control inflation factor. (TIF) [file pgen.1006122.s014.tif]

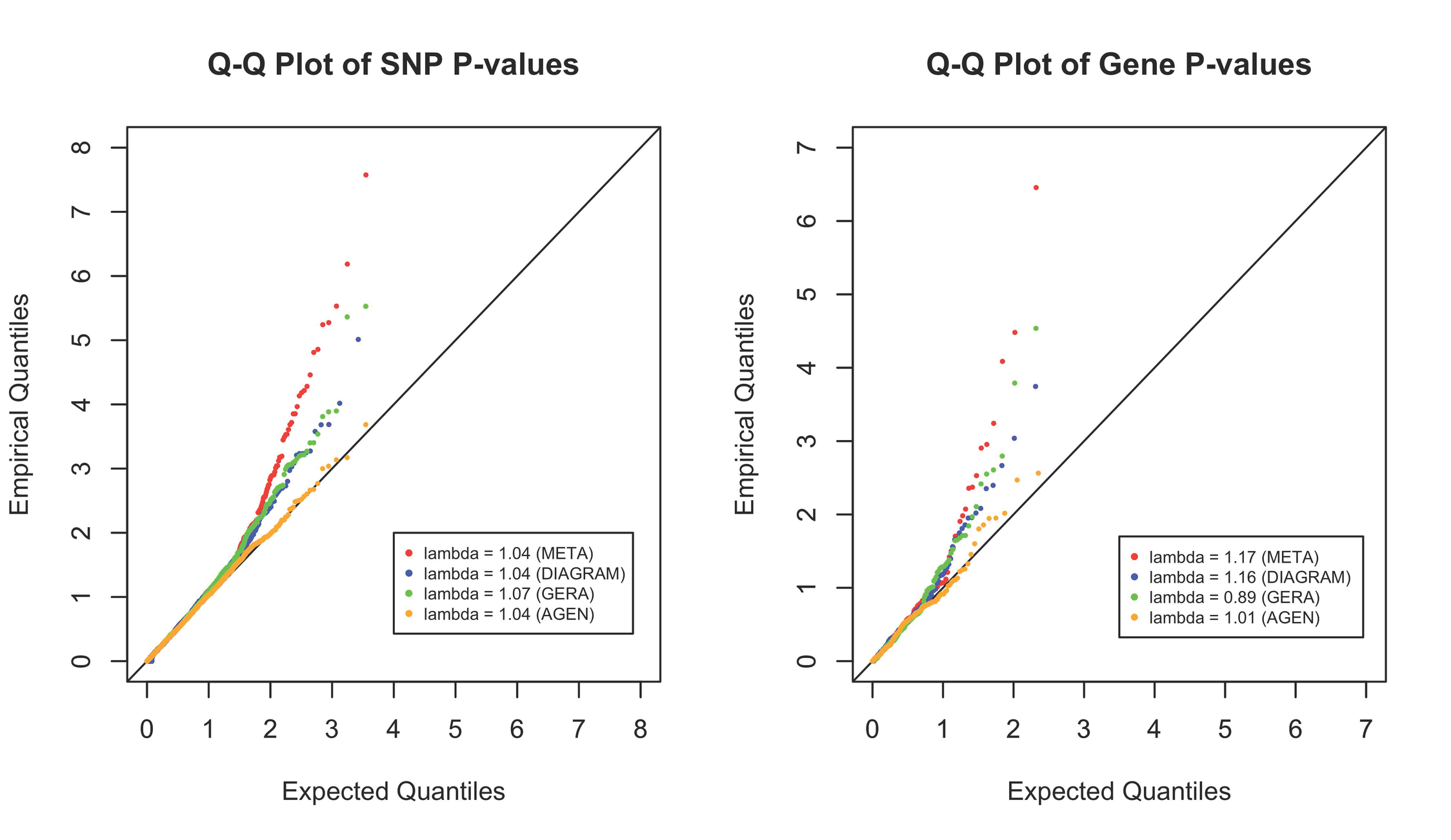

Supplement: S6 Fig — (TIF) [file pgen.1006122.s015.tif]

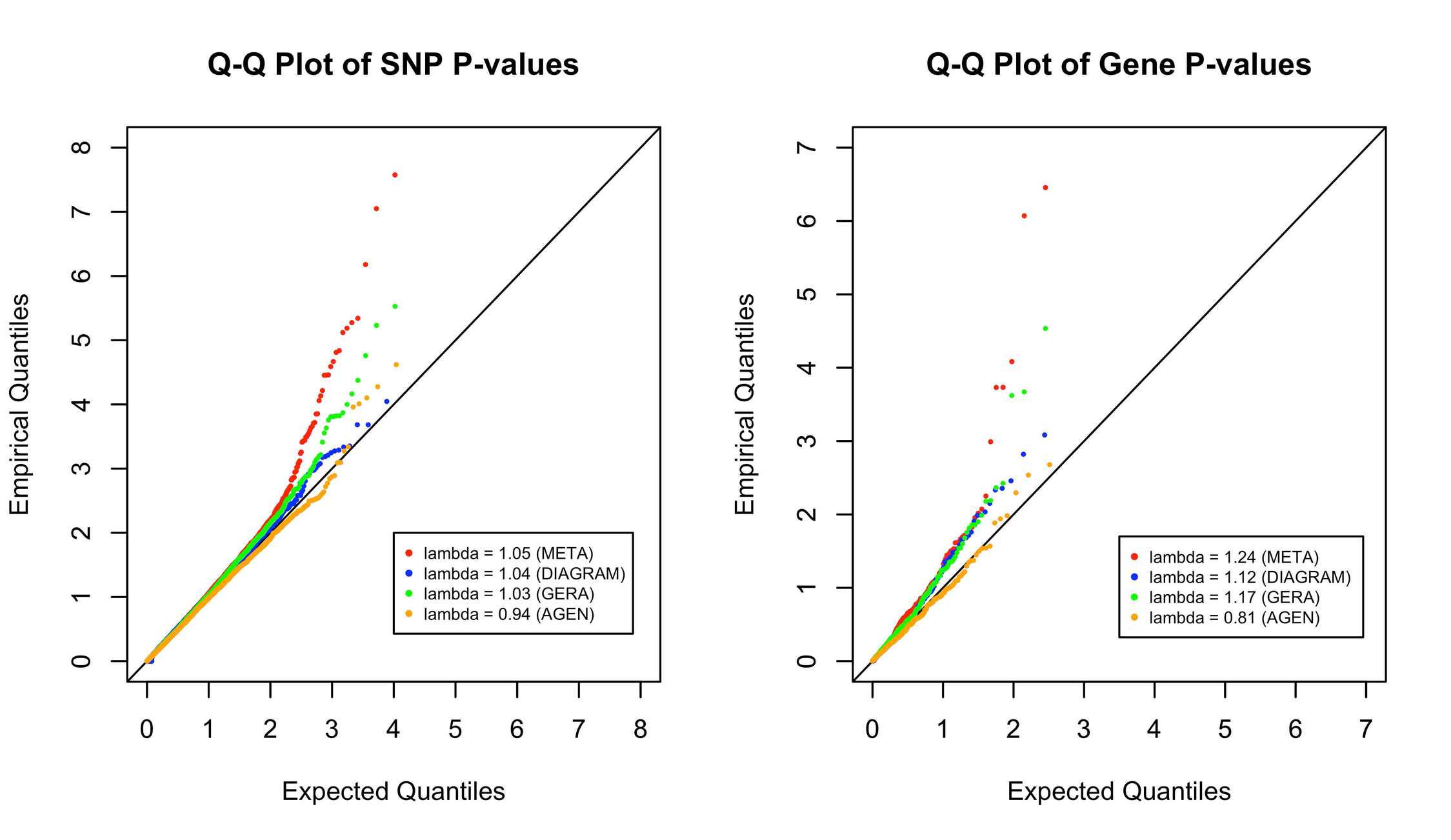

Supplement: S7 Fig — (TIF) [file pgen.1006122.s016.tif]

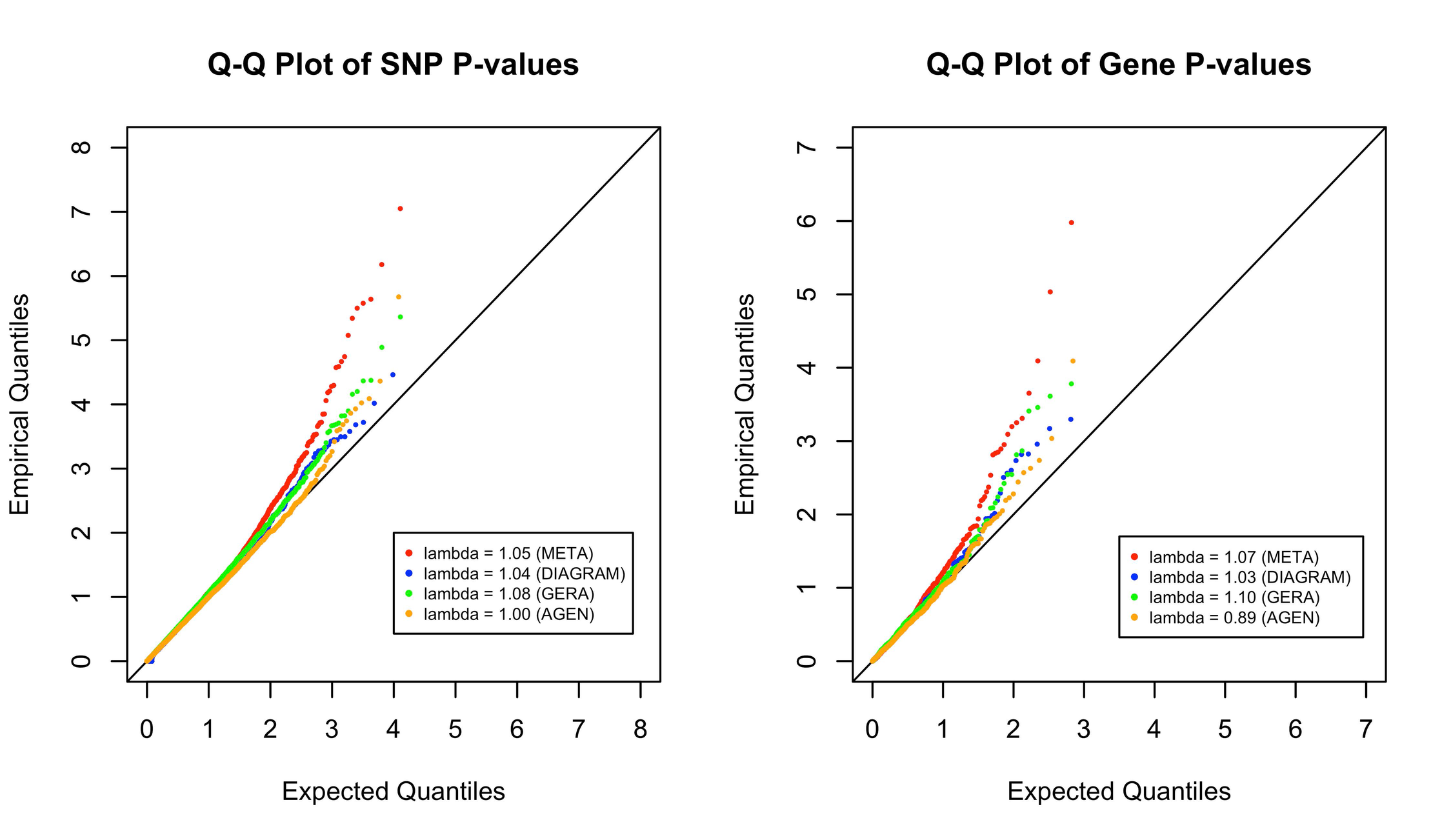

Supplement: S8 Fig — (TIF) [file pgen.1006122.s017.tif]

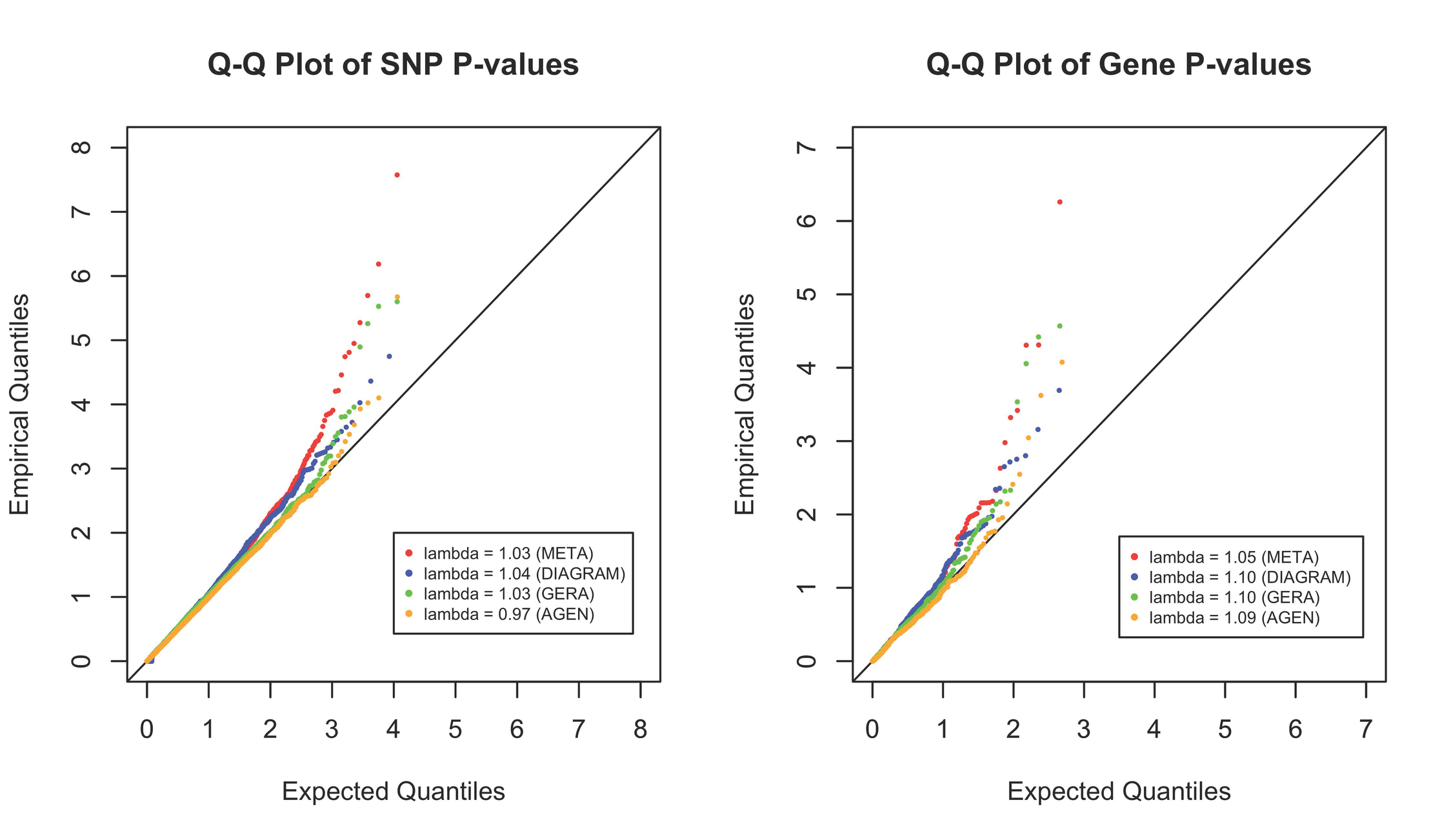

Supplement: S9 Fig — (TIF) [file pgen.1006122.s018.tif]

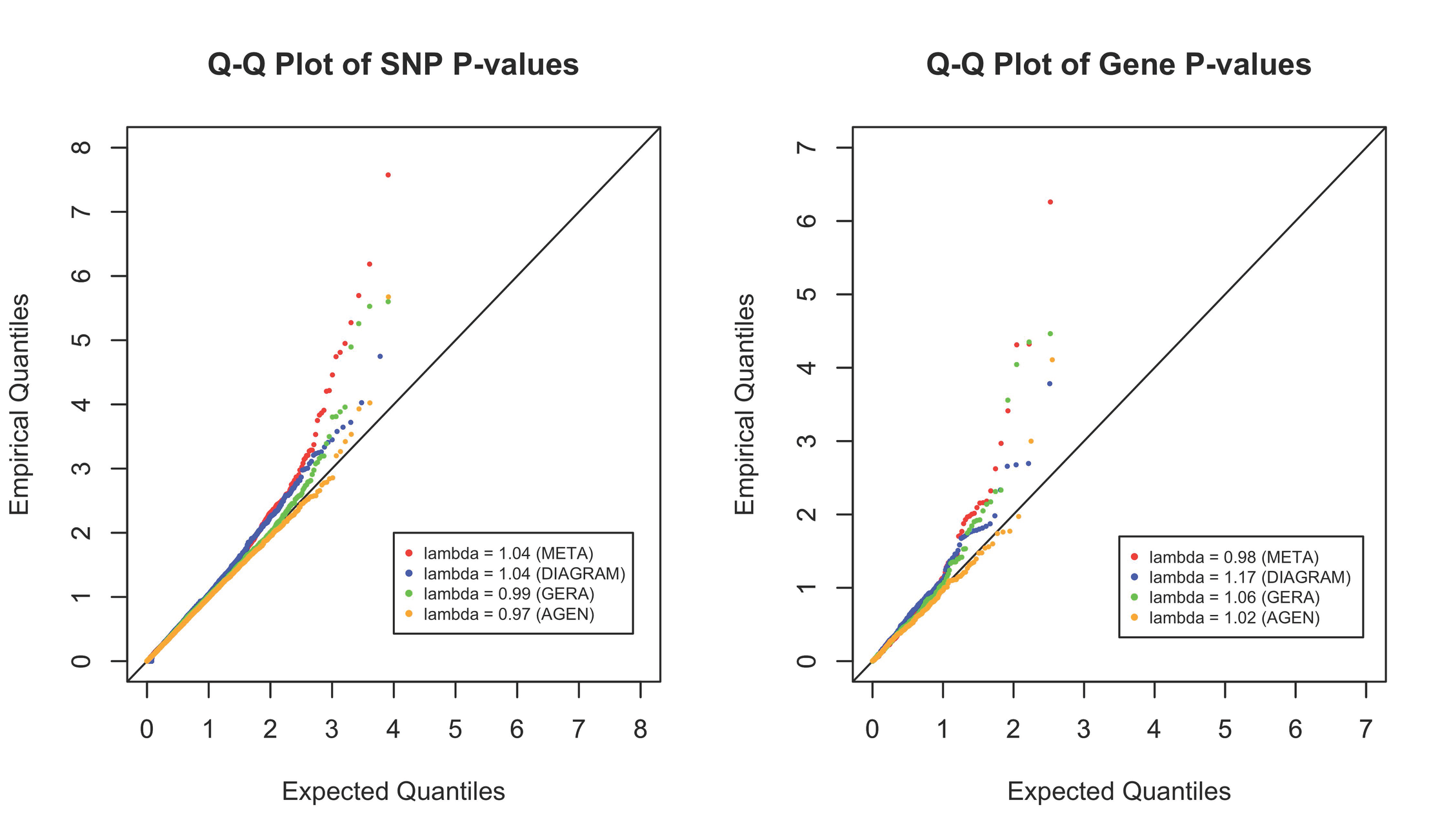

Supplement: S10 Fig — (TIF) [file pgen.1006122.s019.tif]

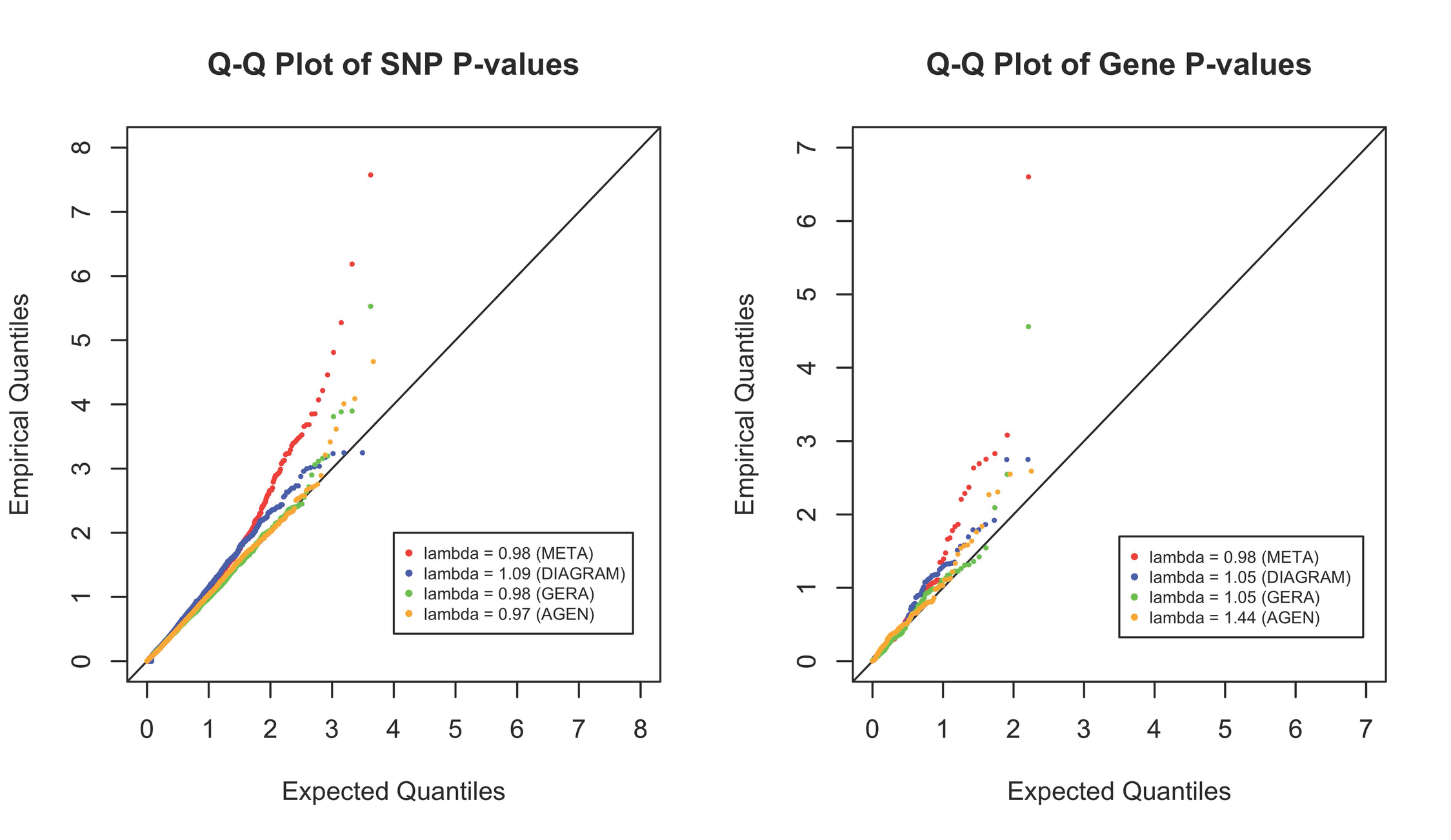

Supplement: S11 Fig — (TIF) [file pgen.1006122.s020.tif]

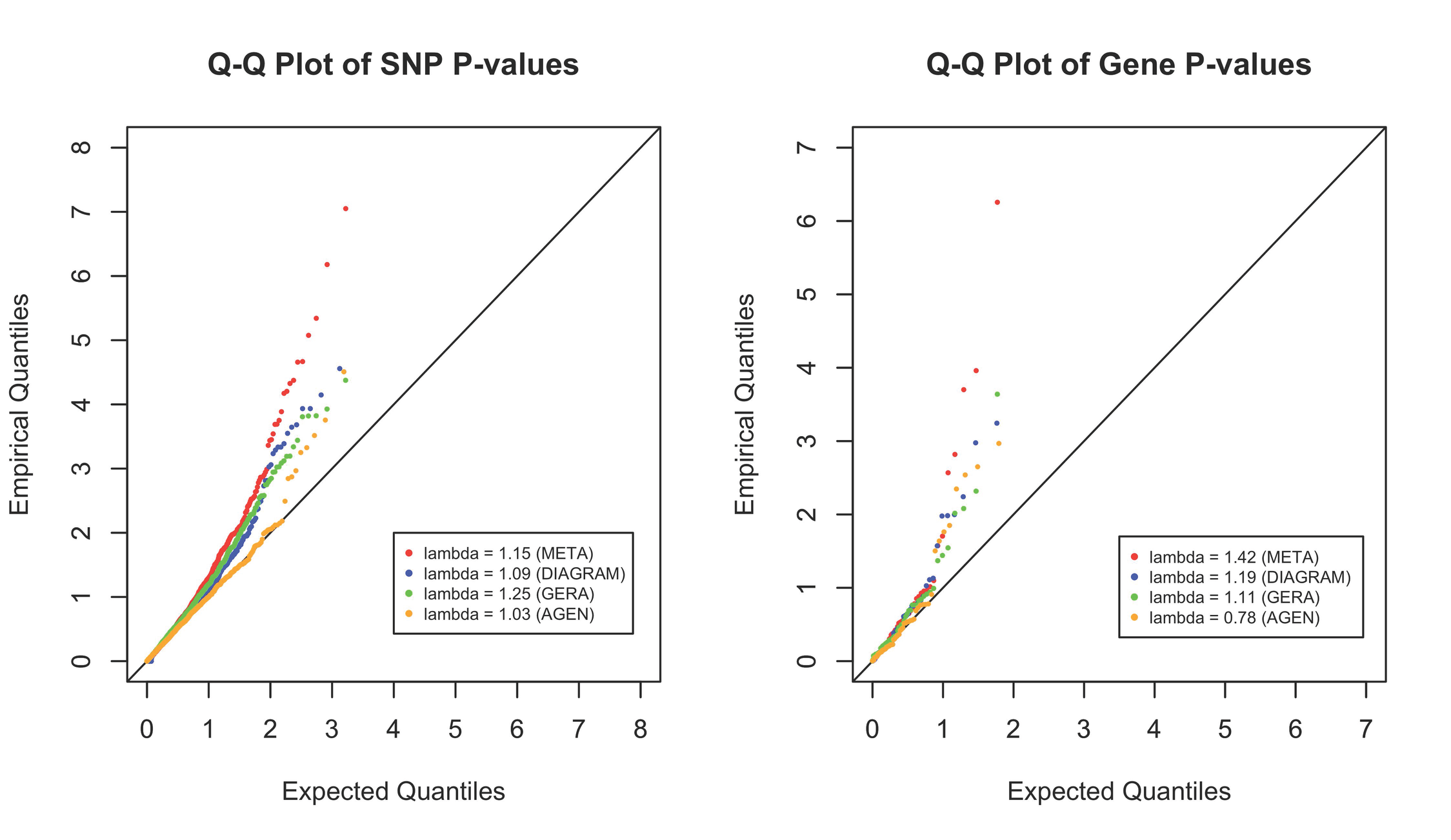

Supplement: S12 Fig — (TIF) [file pgen.1006122.s021.tif]

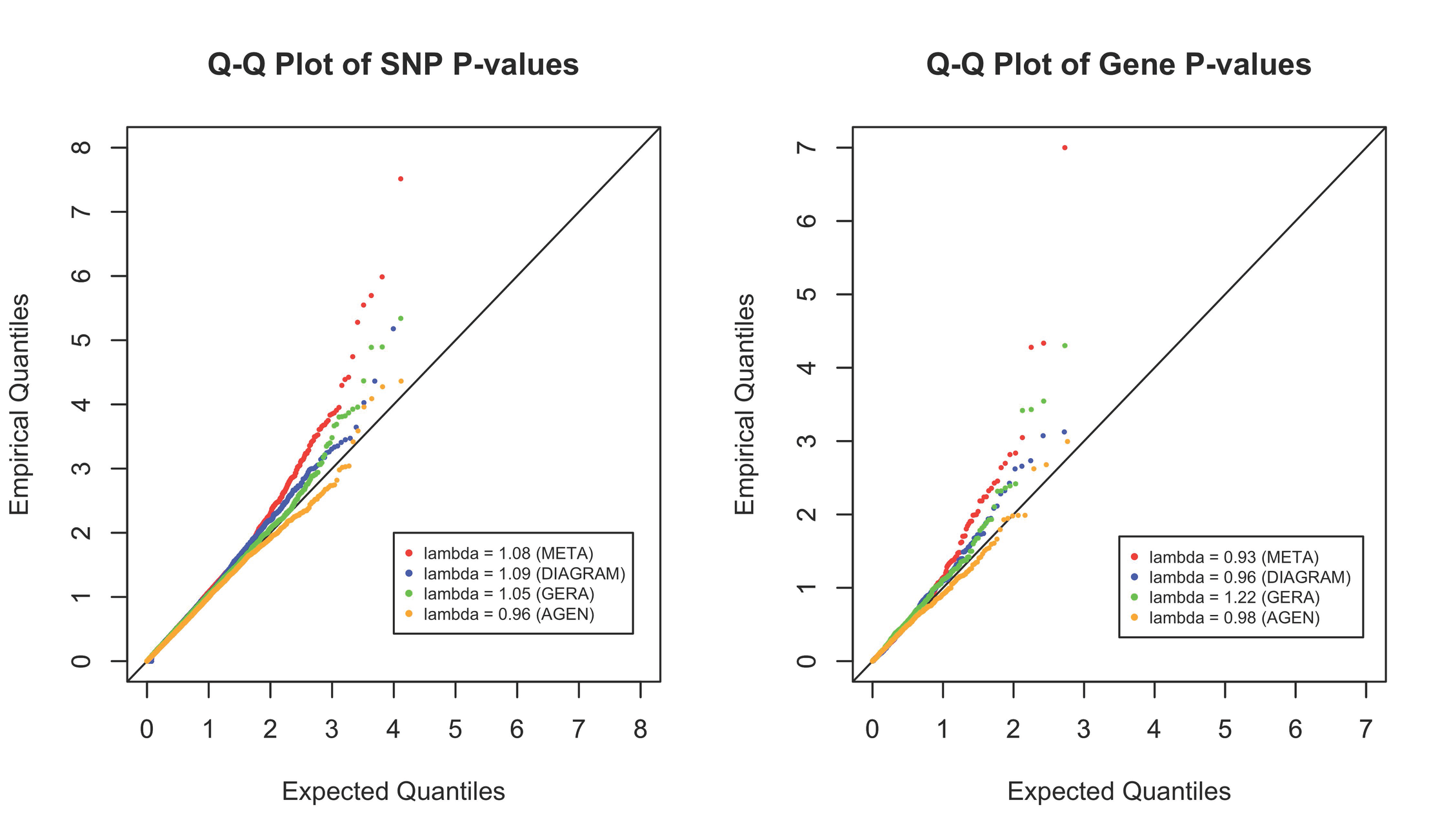

Supplement: S13 Fig — (TIF) [file pgen.1006122.s022.tif]

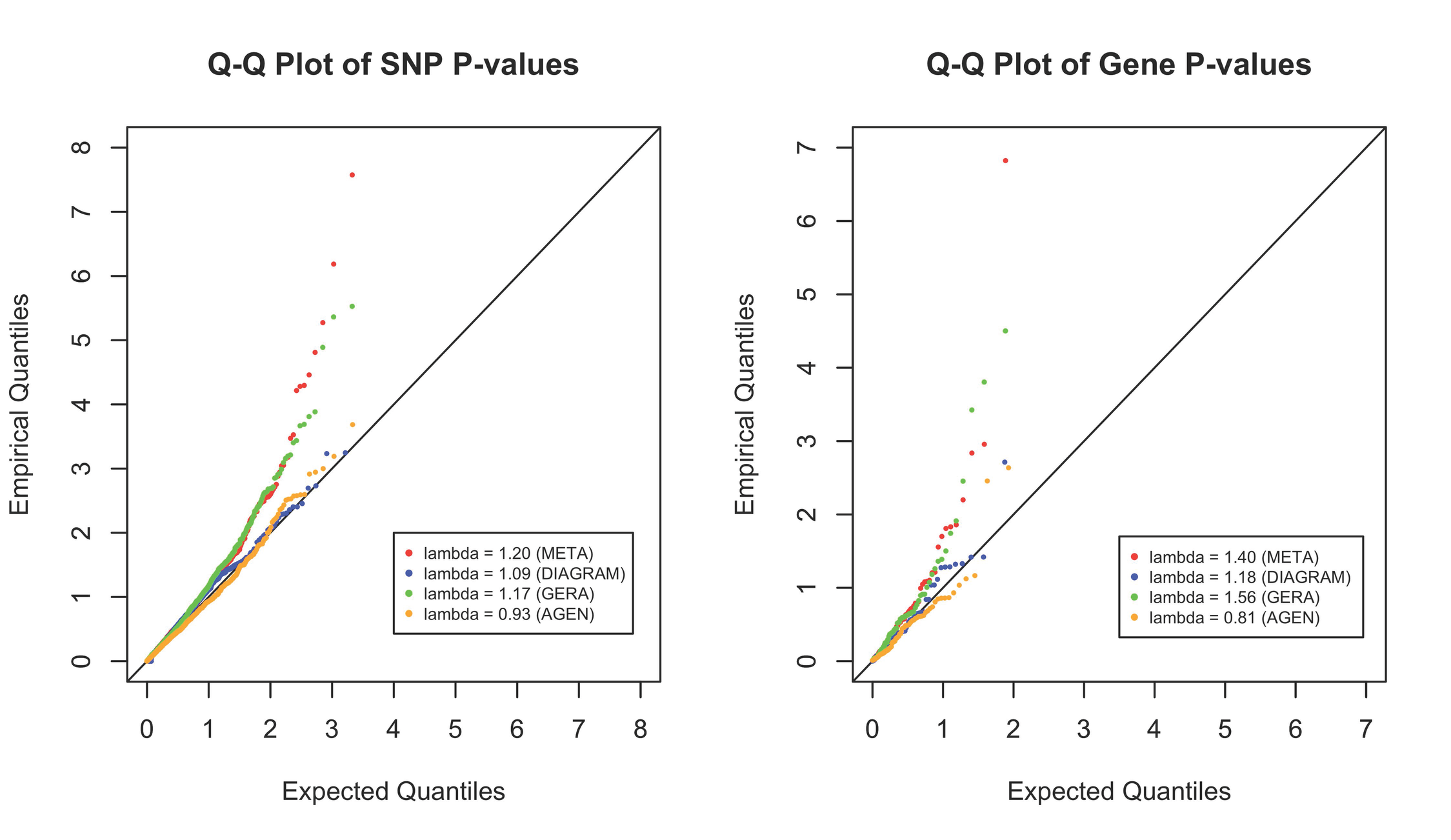

Supplement: S14 Fig — (TIF) [file pgen.1006122.s023.tif]

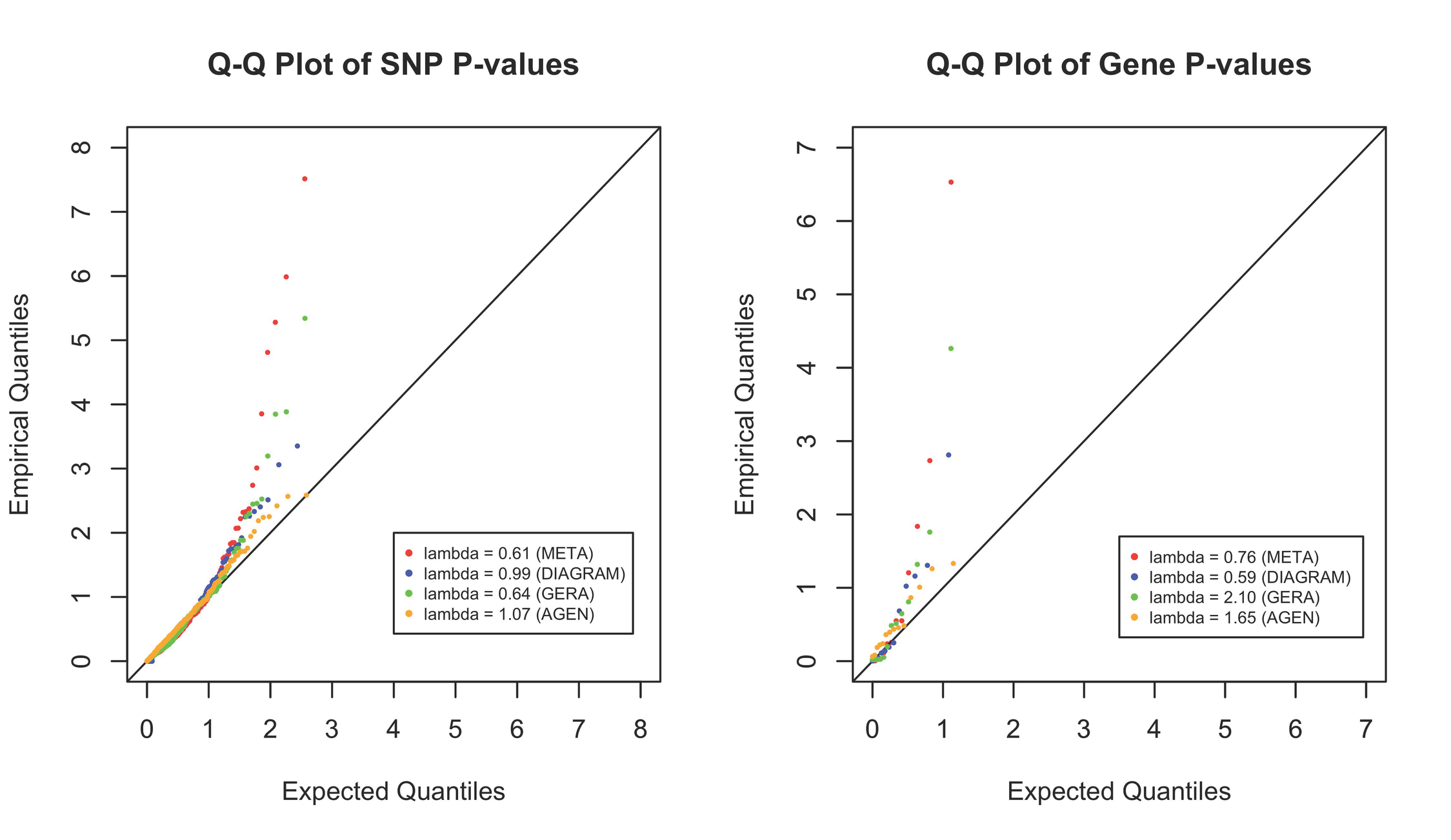

Supplement: S15 Fig — (TIF) [file pgen.1006122.s024.tif]

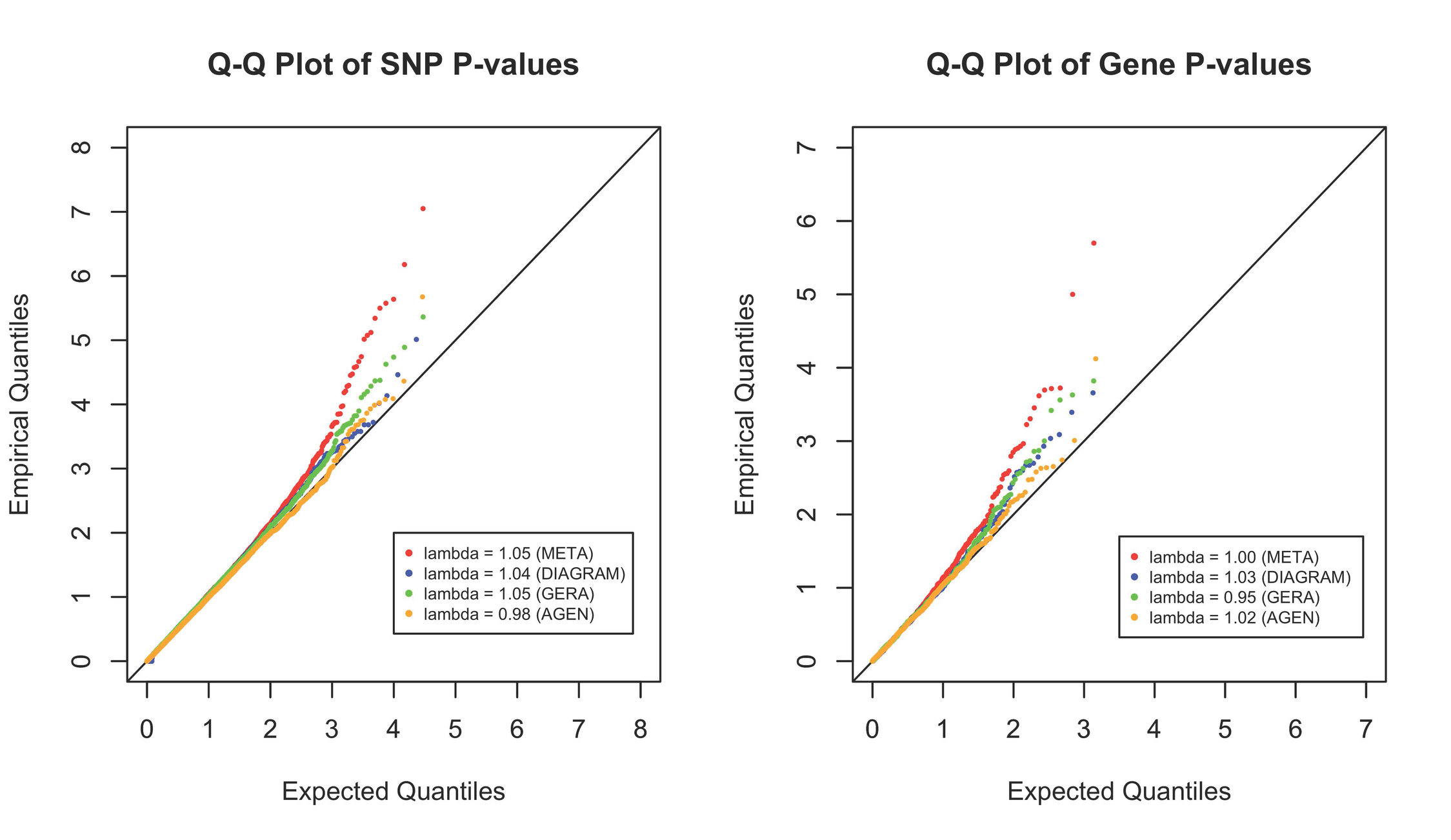

Supplement: S16 Fig — (TIF) [file pgen.1006122.s025.tif]

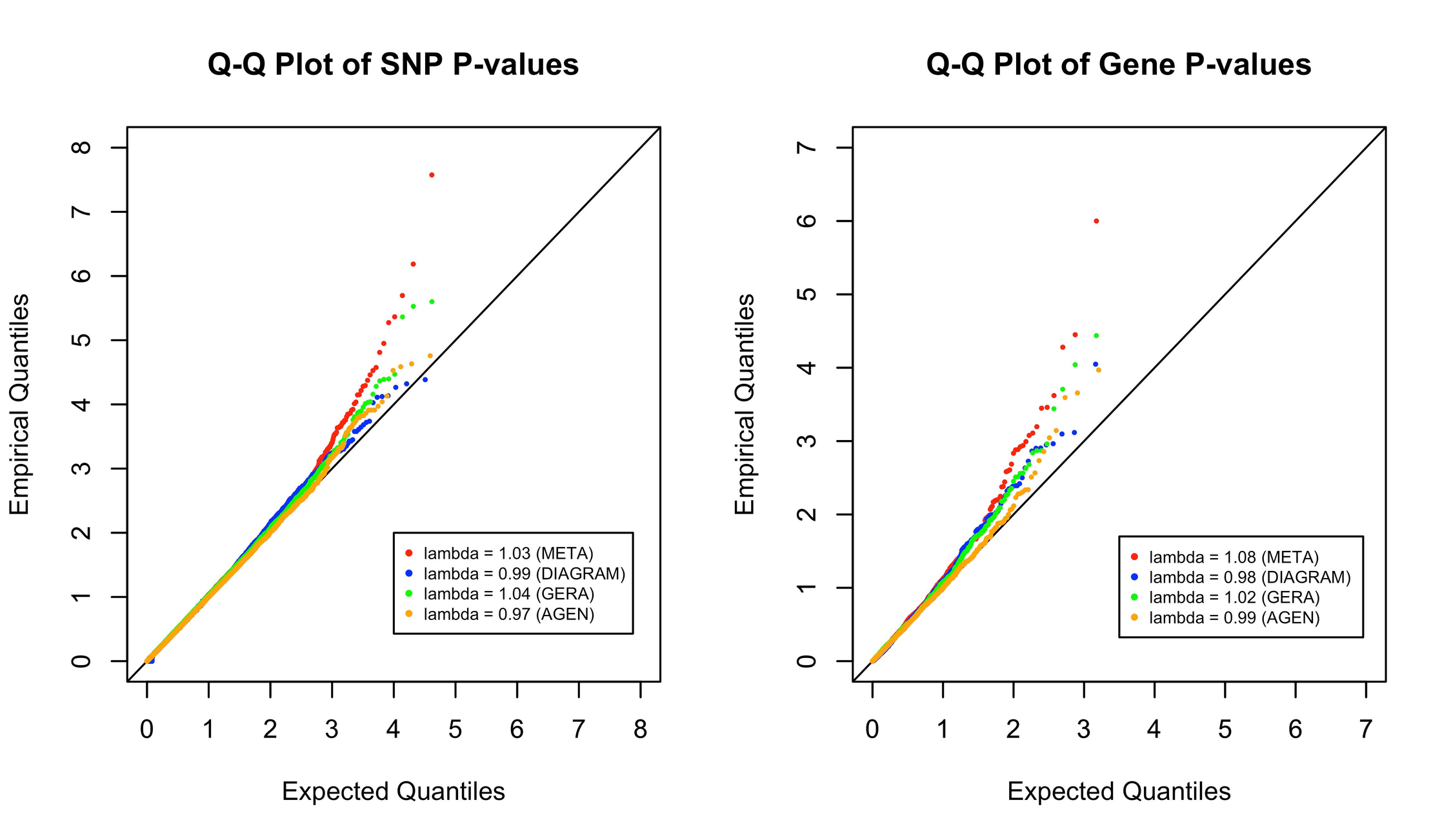

Supplement: S17 Fig — (TIF) [file pgen.1006122.s026.tif]

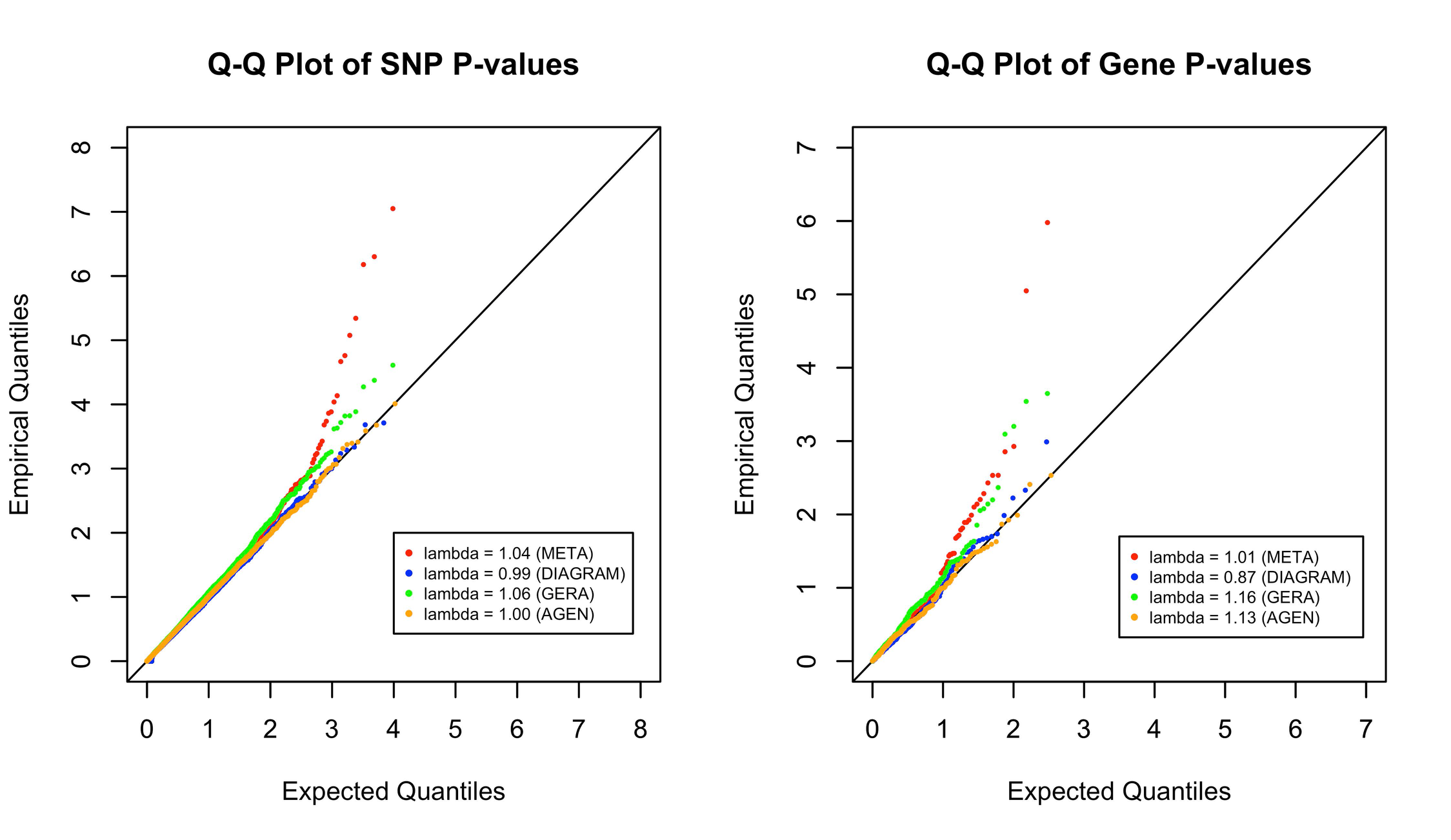

Supplement: S18 Fig — (TIF) [file pgen.1006122.s027.tif]

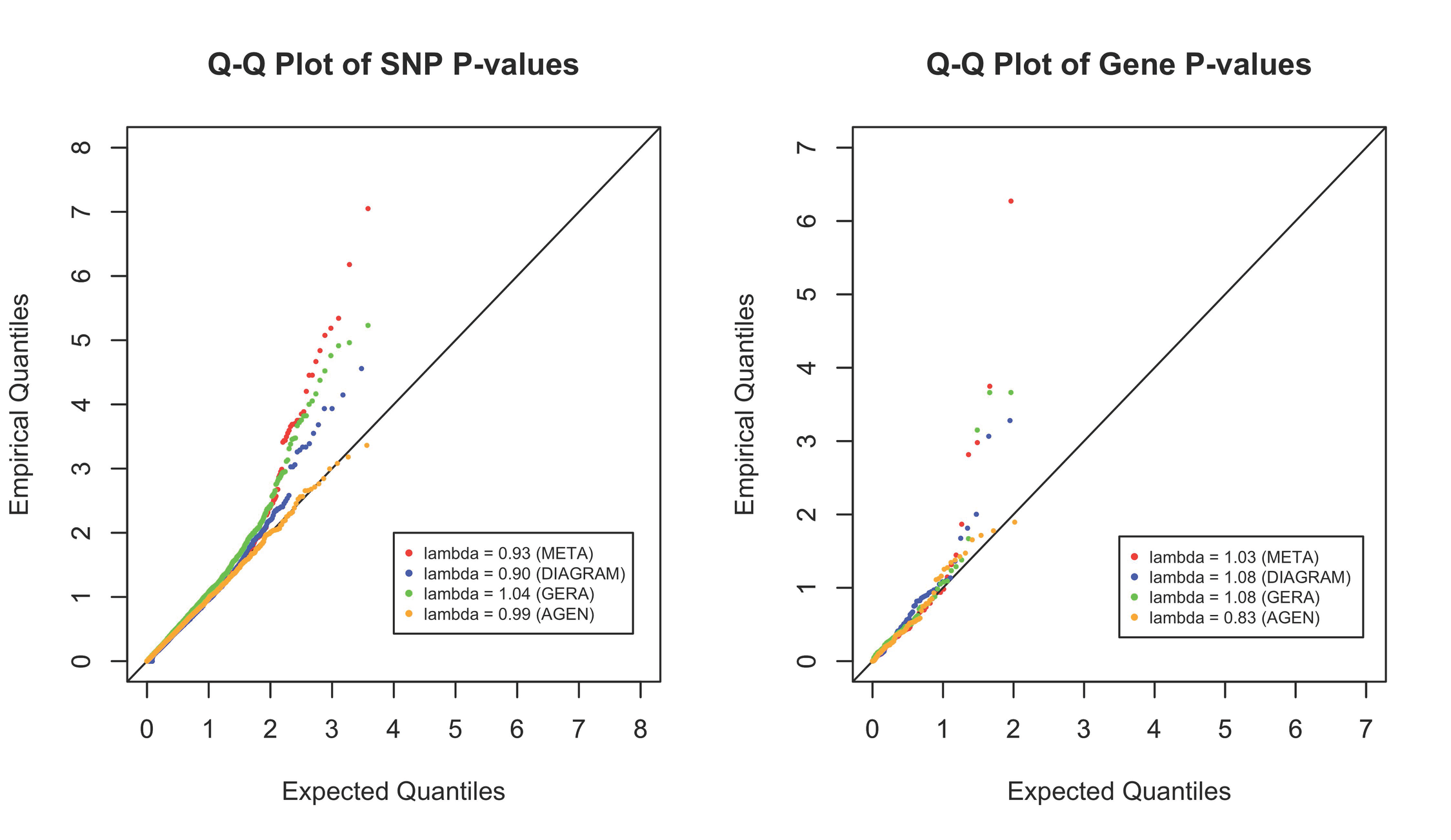

Supplement: S19 Fig — (TIF) [file pgen.1006122.s028.tif]

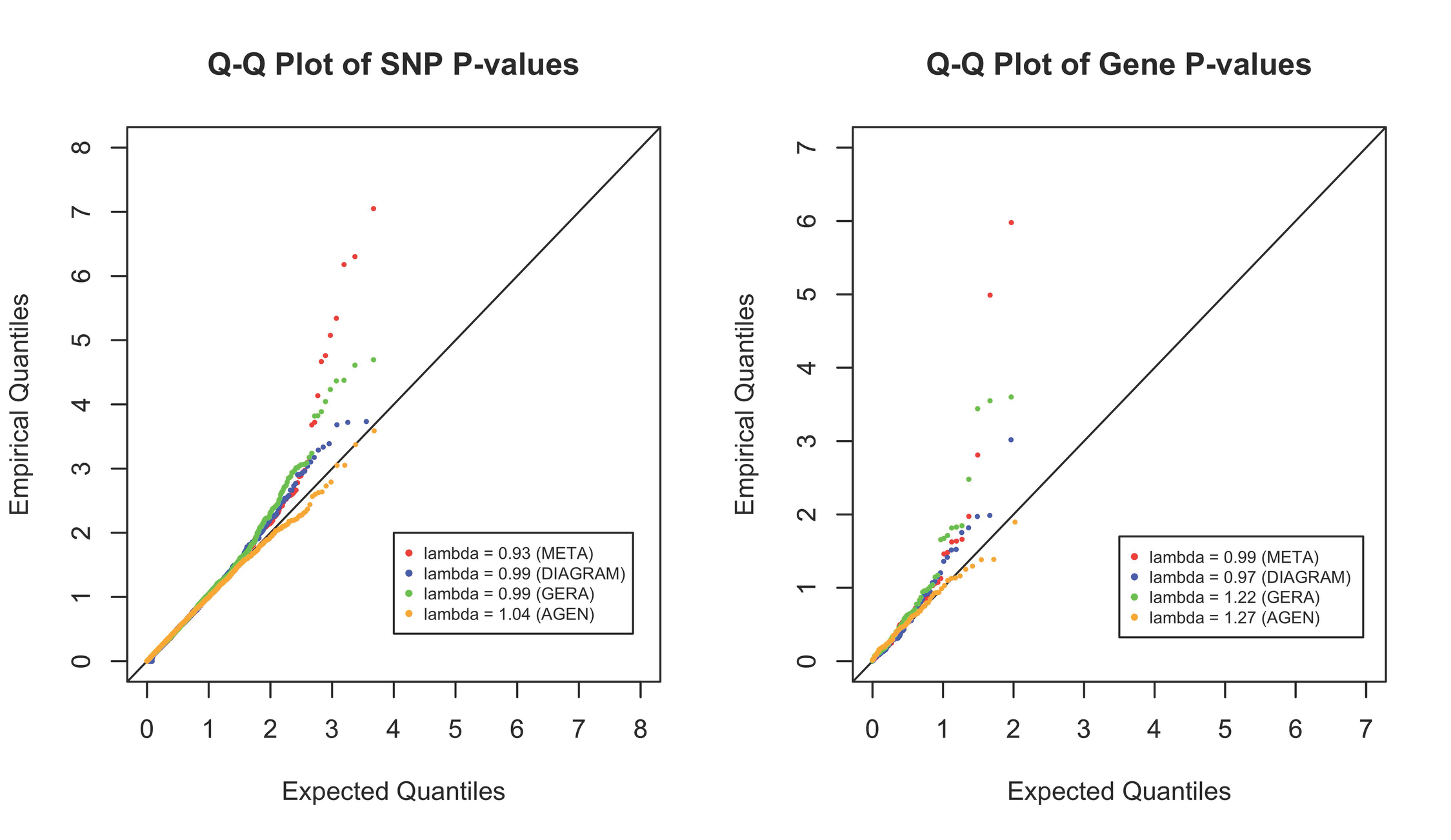

Supplement: S20 Fig — (TIF) [file pgen.1006122.s029.tif]

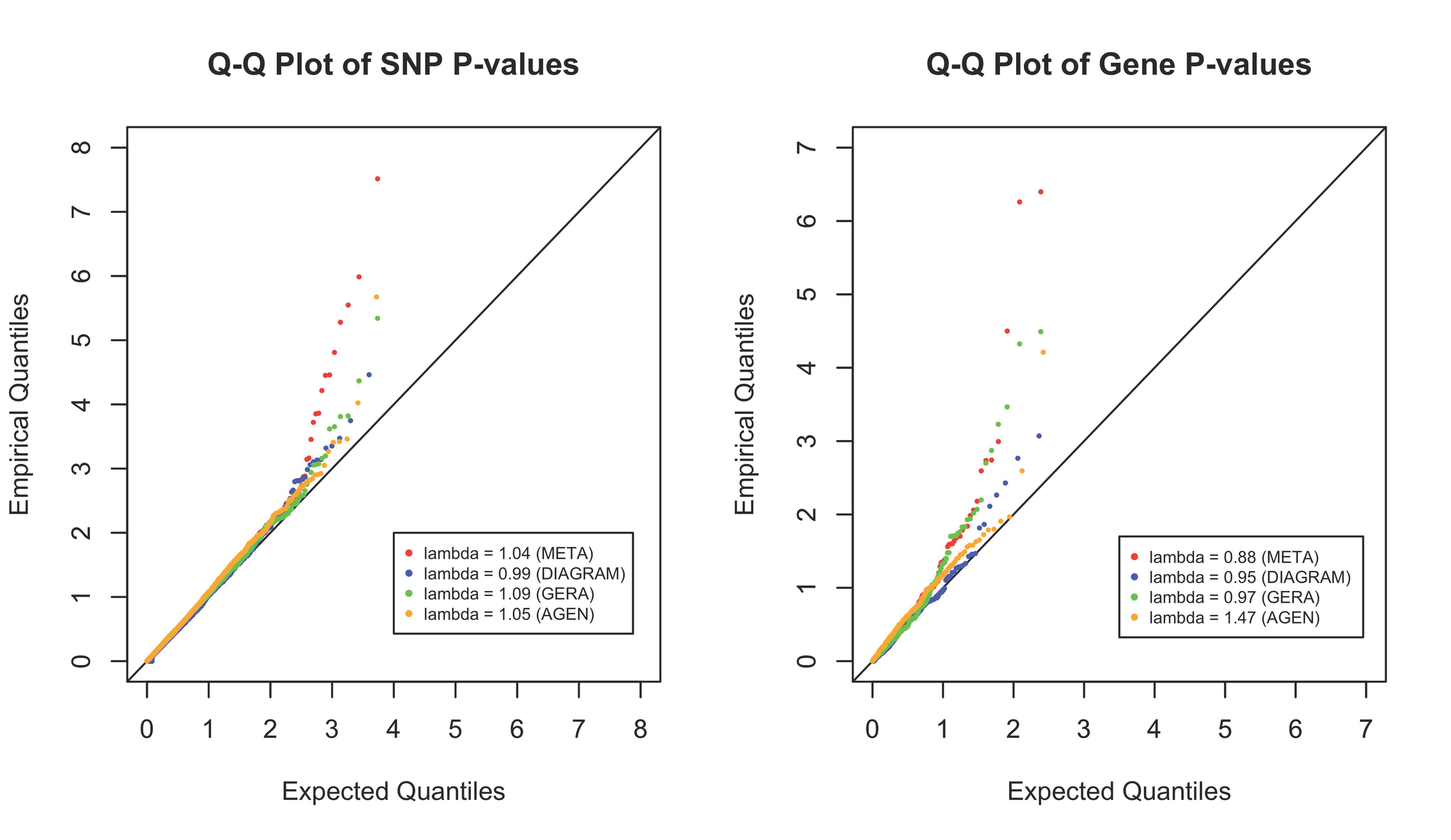

Supplement: S21 Fig — (TIF) [file pgen.1006122.s030.tif]

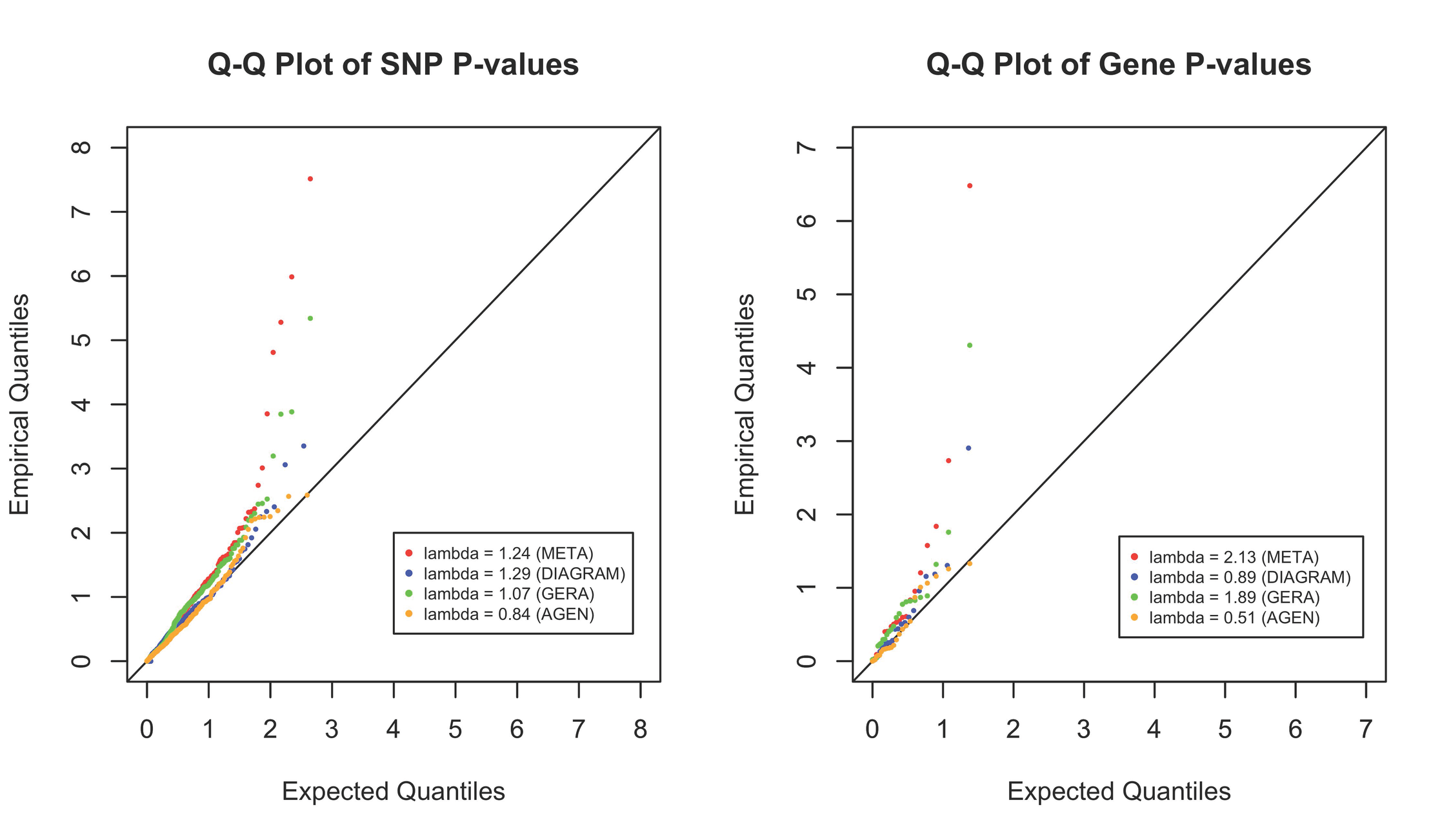

Supplement: S22 Fig — (TIF) [file pgen.1006122.s031.tif]

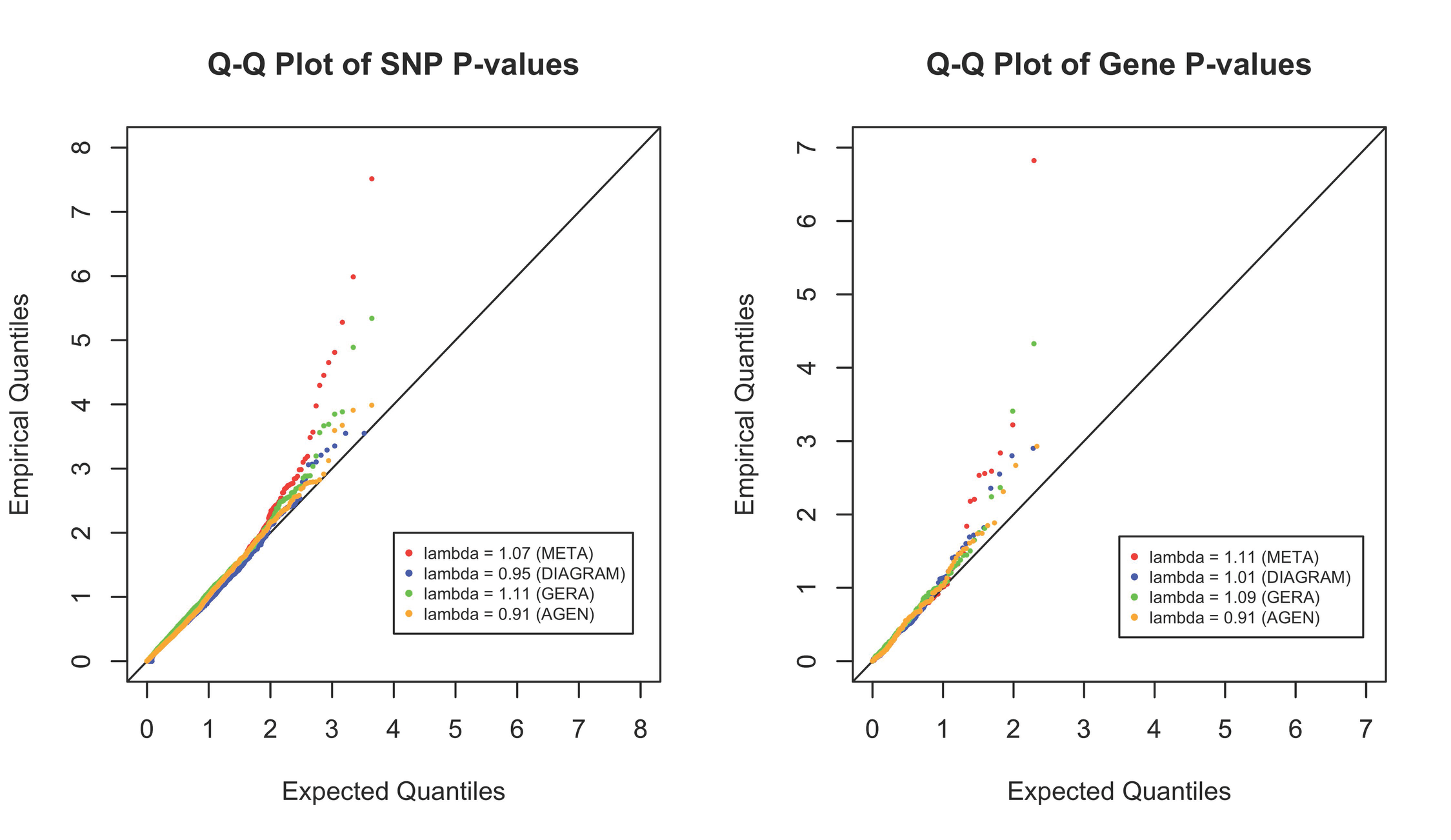

Supplement: S23 Fig — (TIF) [file pgen.1006122.s032.tif]

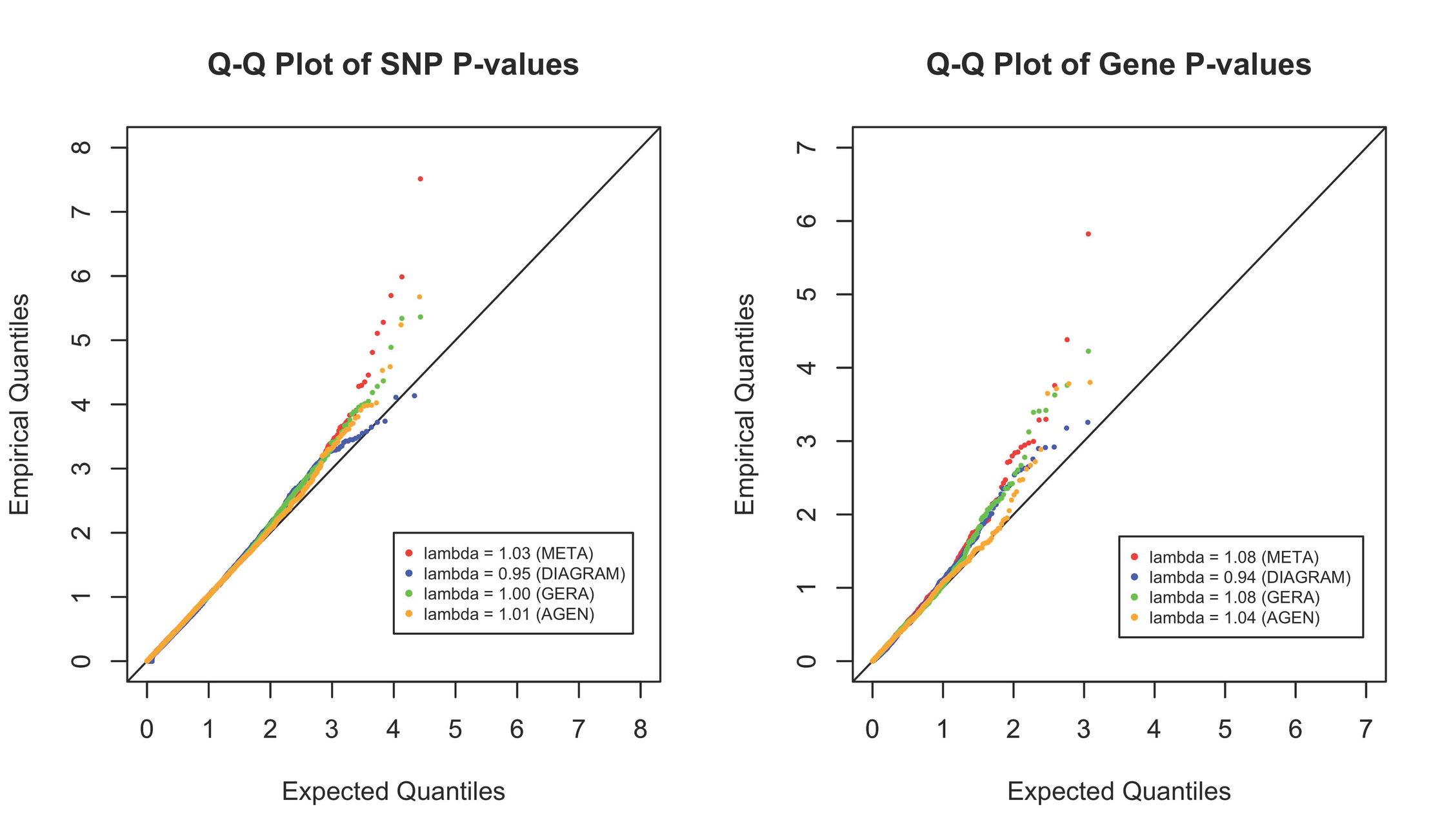

Supplement: S24 Fig — (TIF) [file pgen.1006122.s033.tif]

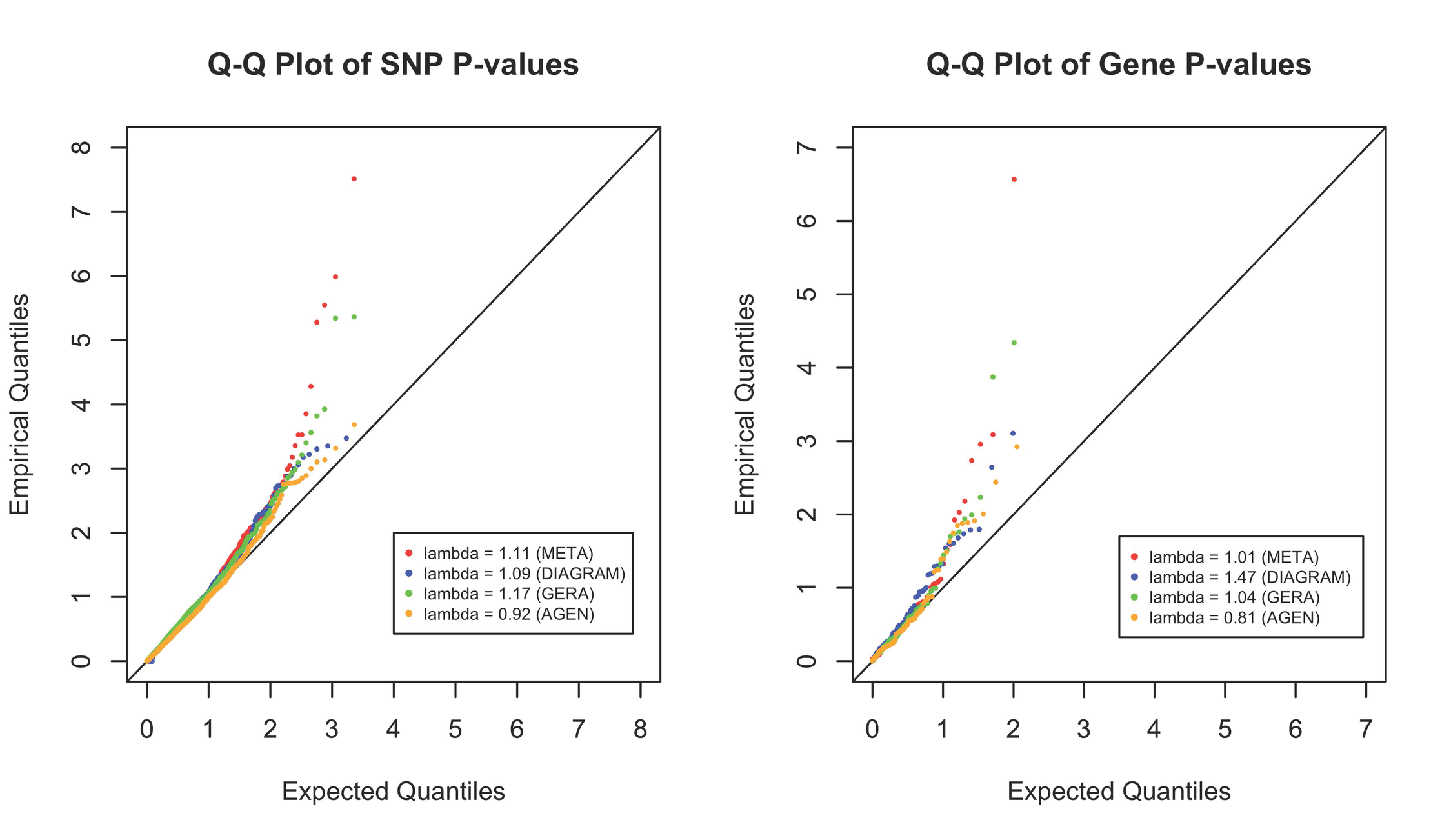

Supplement: S25 Fig — (TIF) [file pgen.1006122.s034.tif]

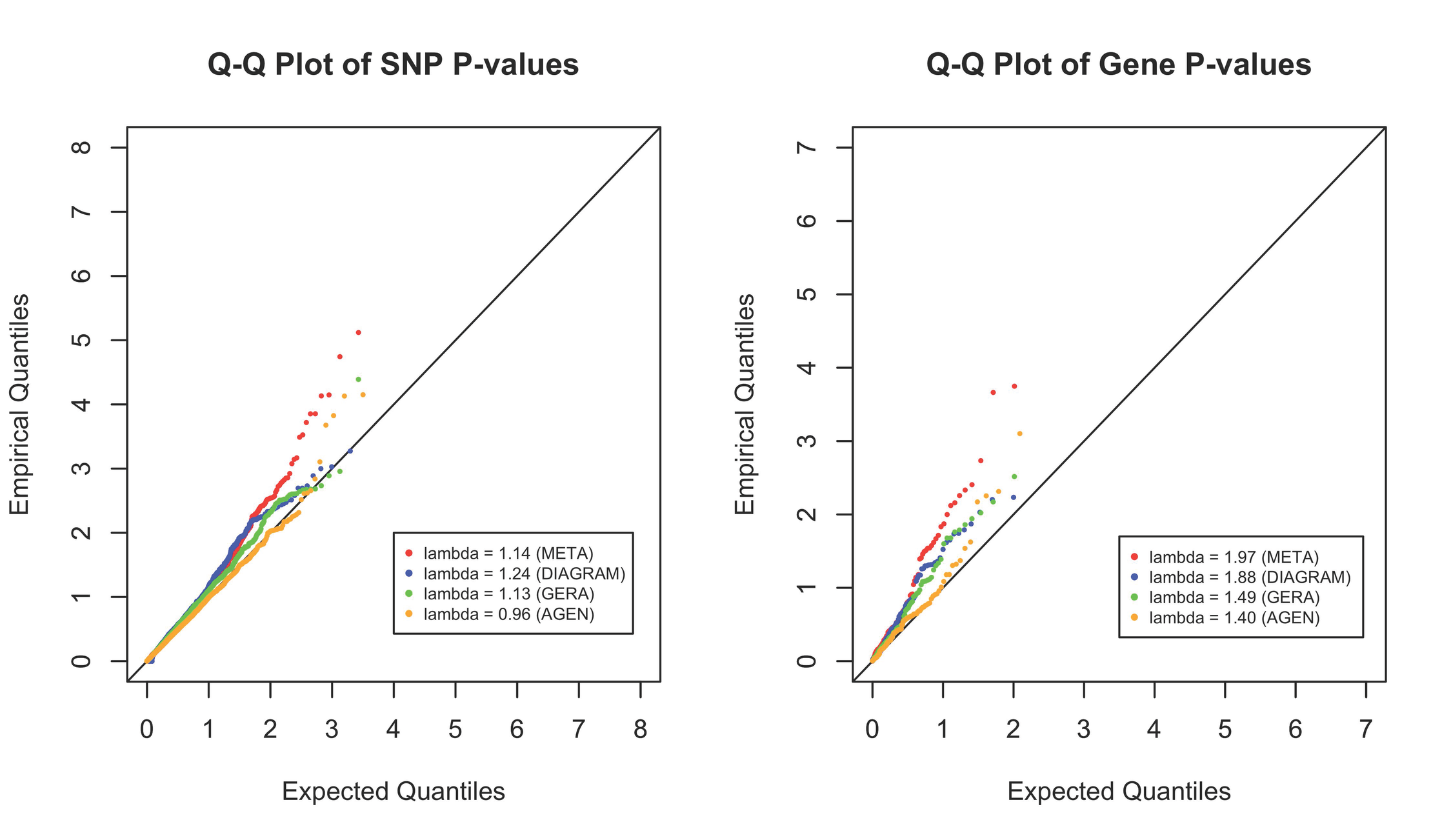

Supplement: S26 Fig — (TIF) [file pgen.1006122.s035.tif]

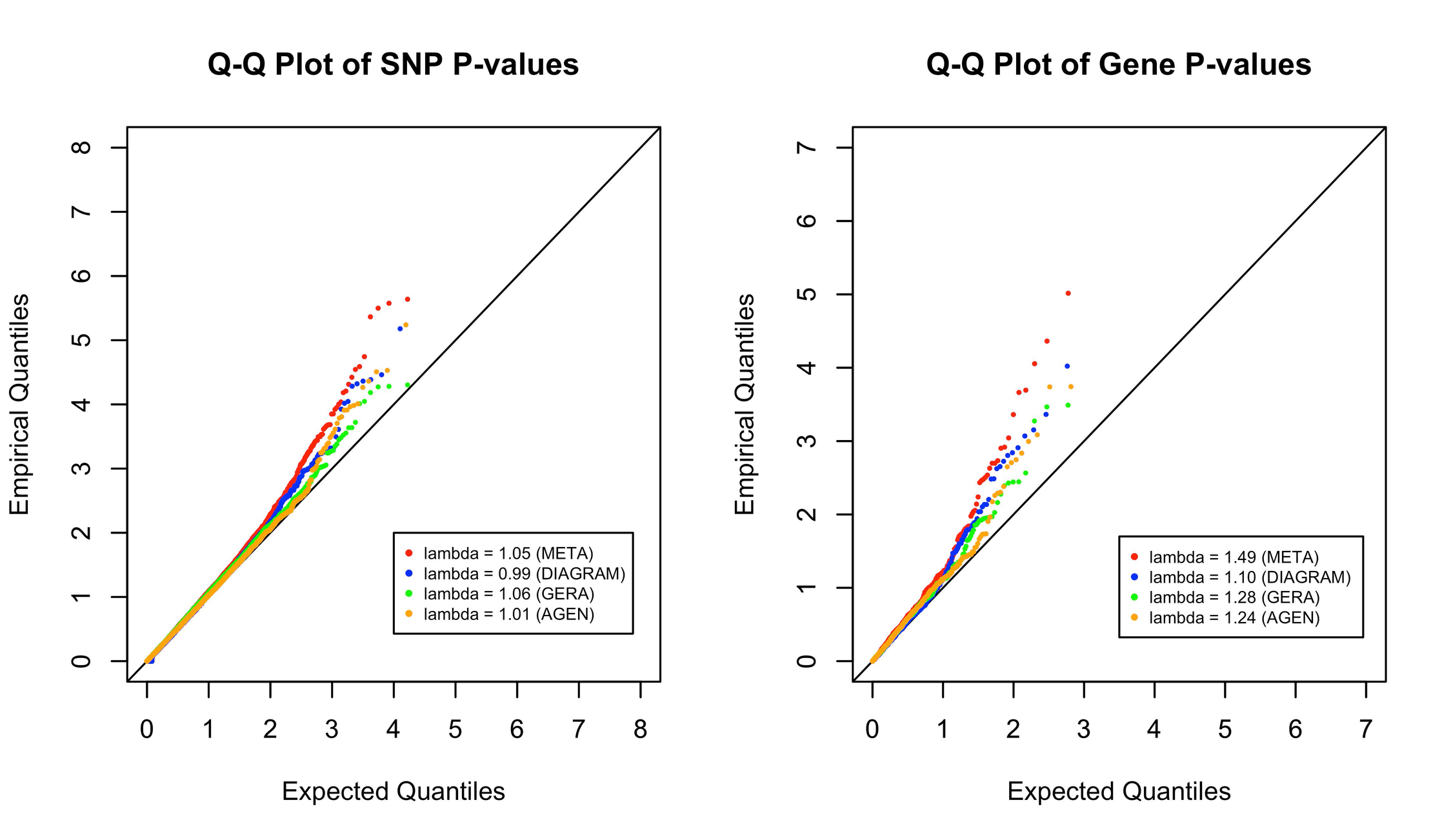

Supplement: S27 Fig — (TIF) [file pgen.1006122.s036.tif]

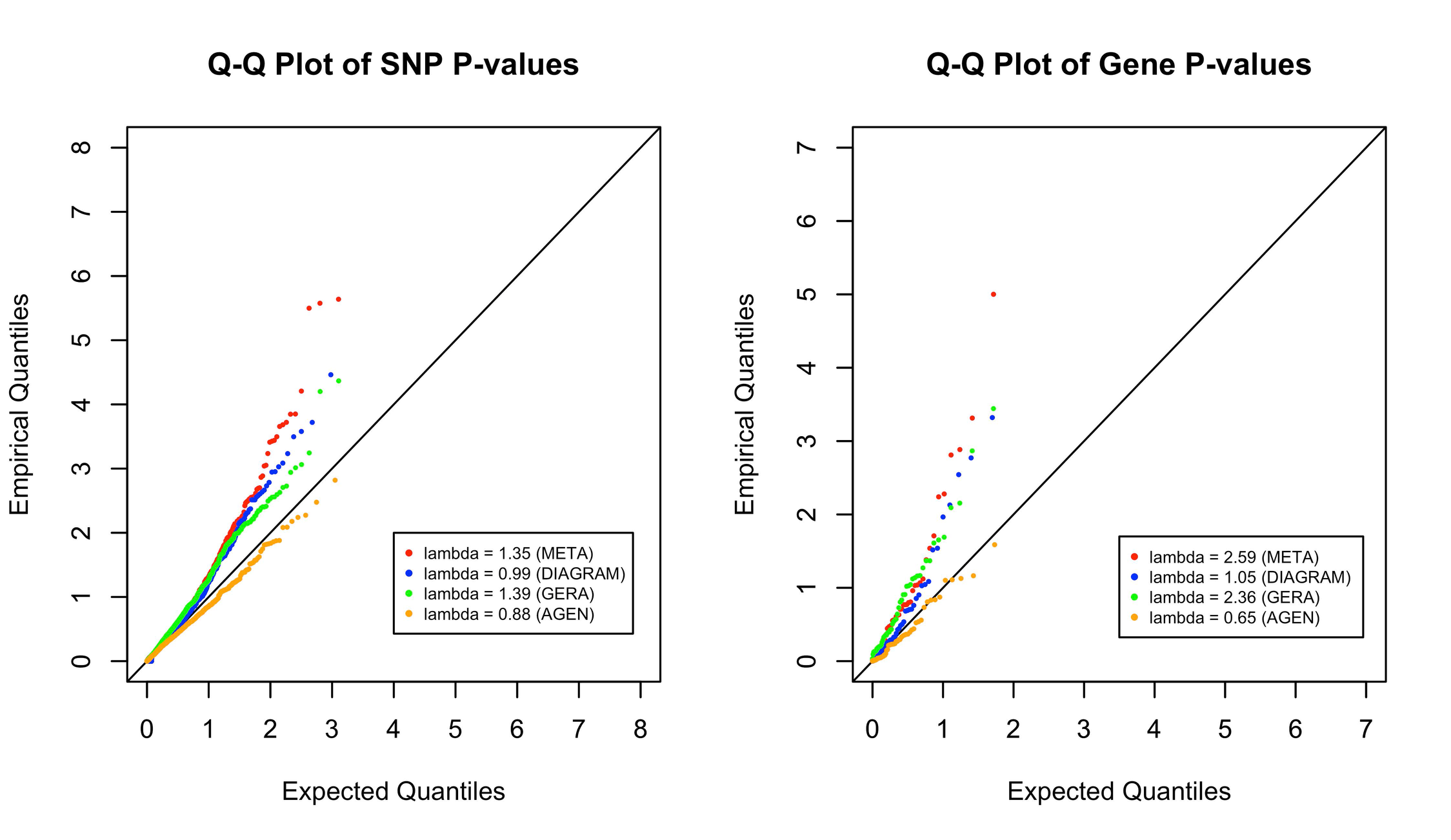

Supplement: S28 Fig — (TIF) [file pgen.1006122.s037.tif]

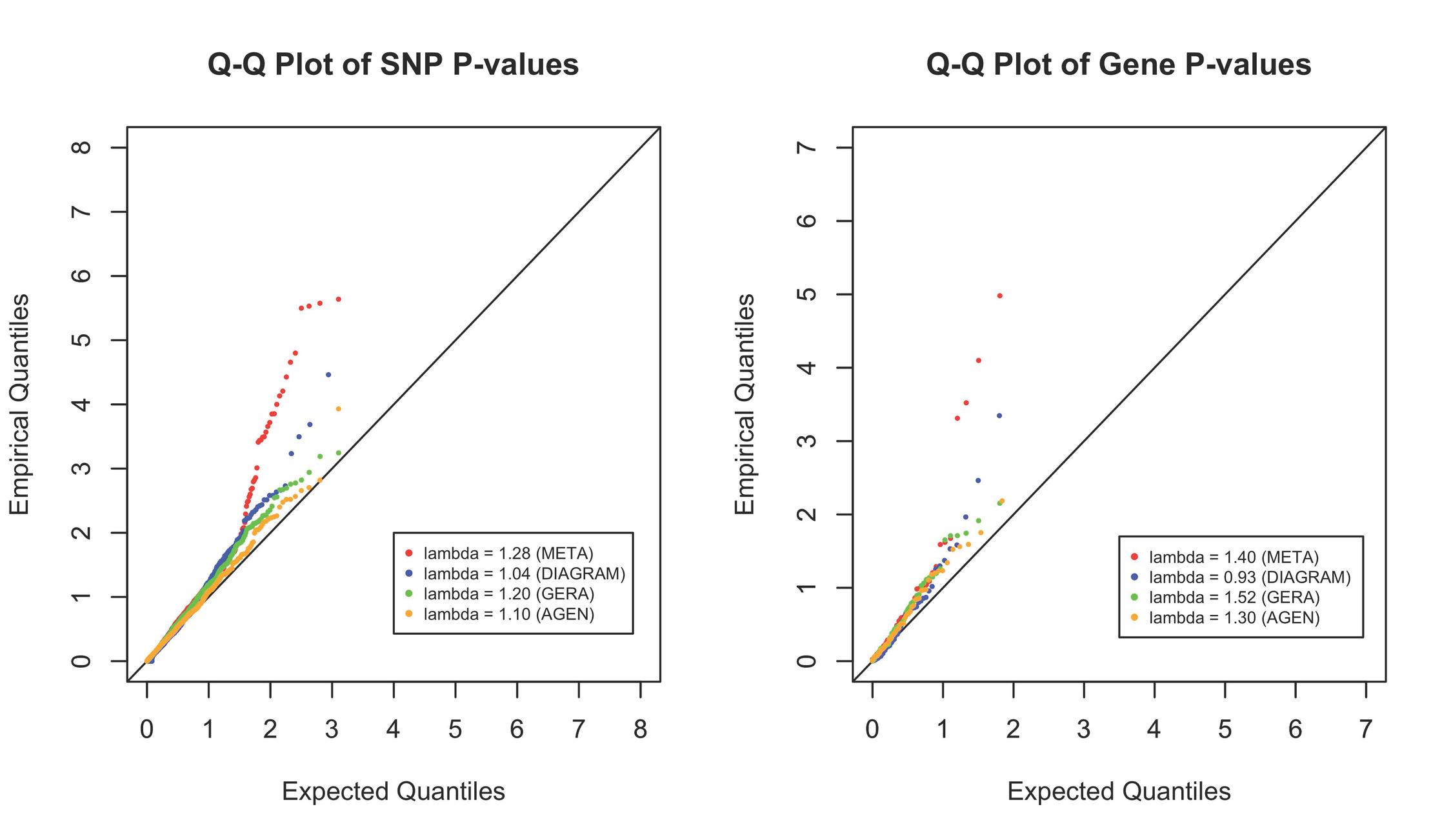

Supplement: S29 Fig — (TIF) [file pgen.1006122.s038.tif]

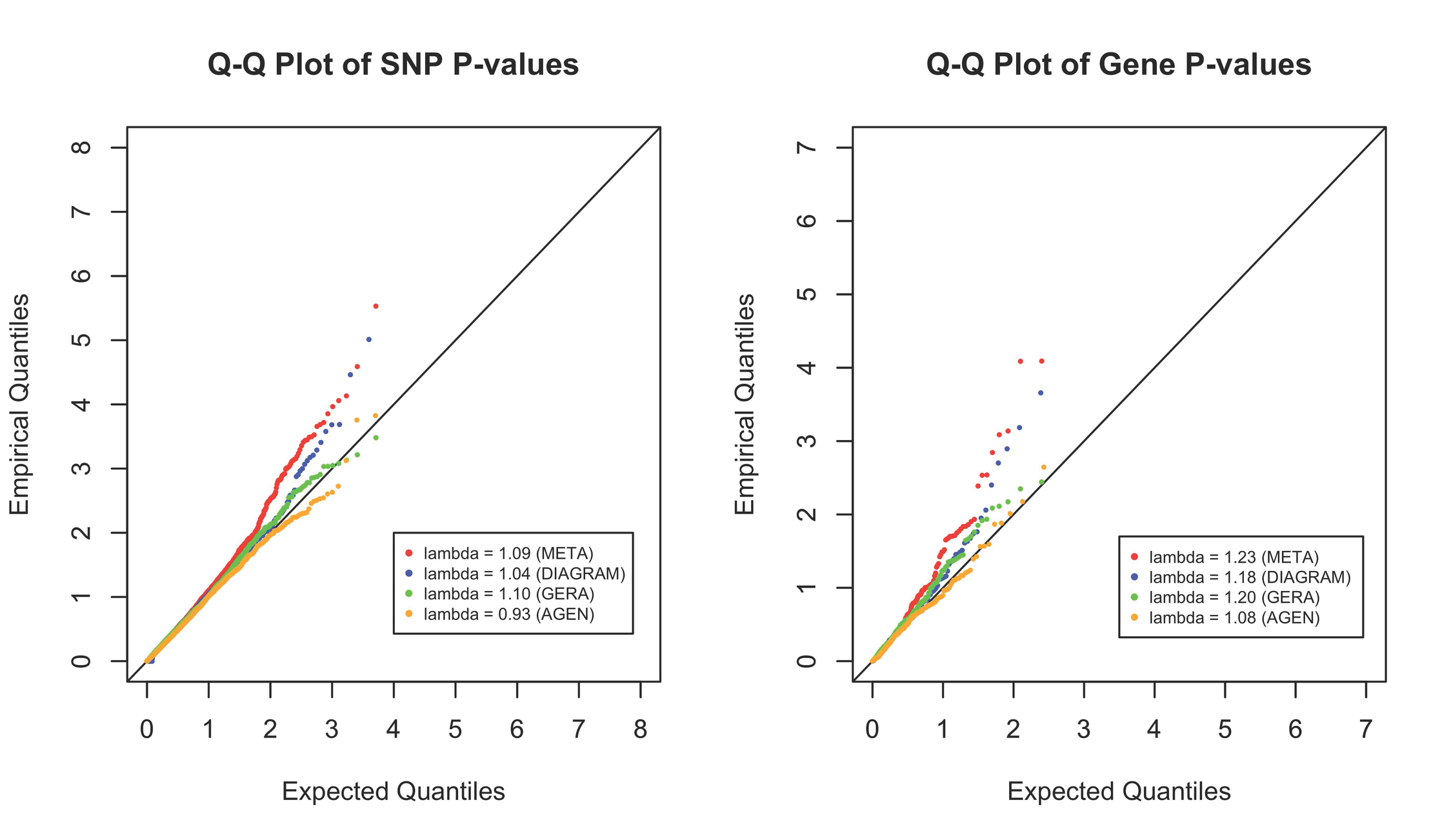

Supplement: S30 Fig — (TIF) [file pgen.1006122.s039.tif]

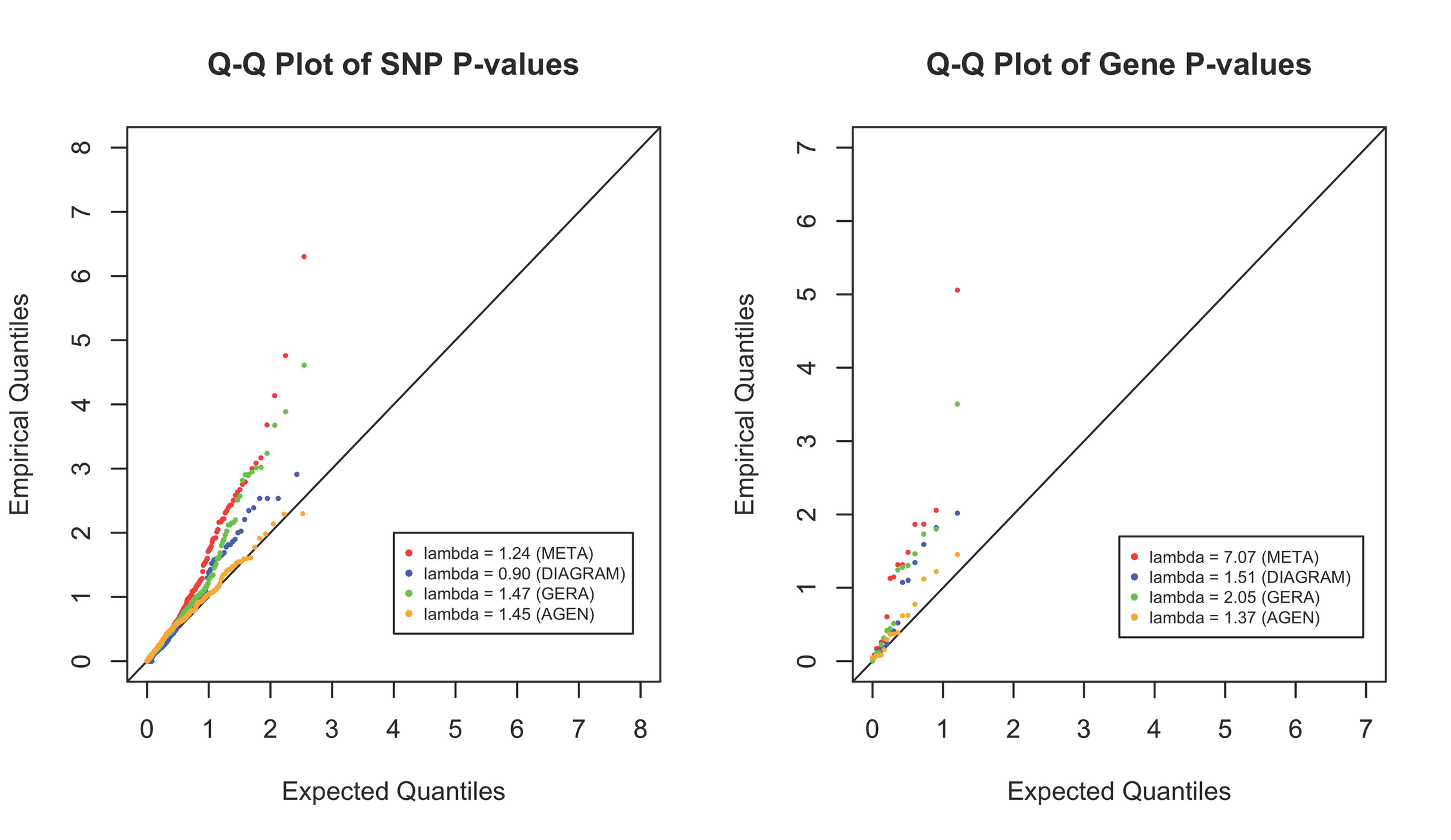

Supplement: S31 Fig — (TIF) [file pgen.1006122.s040.tif]

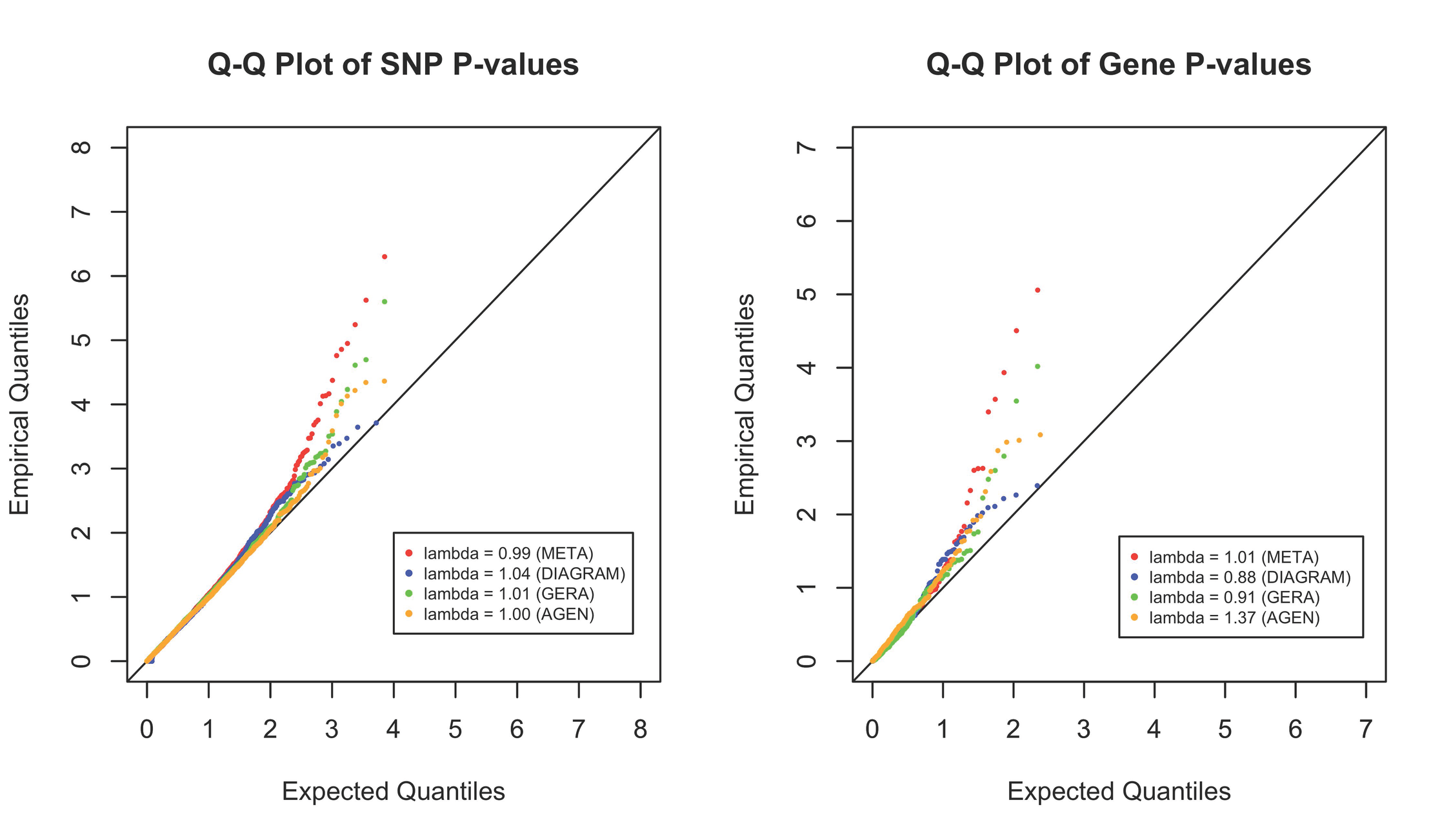

Supplement: S32 Fig — (TIF) [file pgen.1006122.s041.tif]

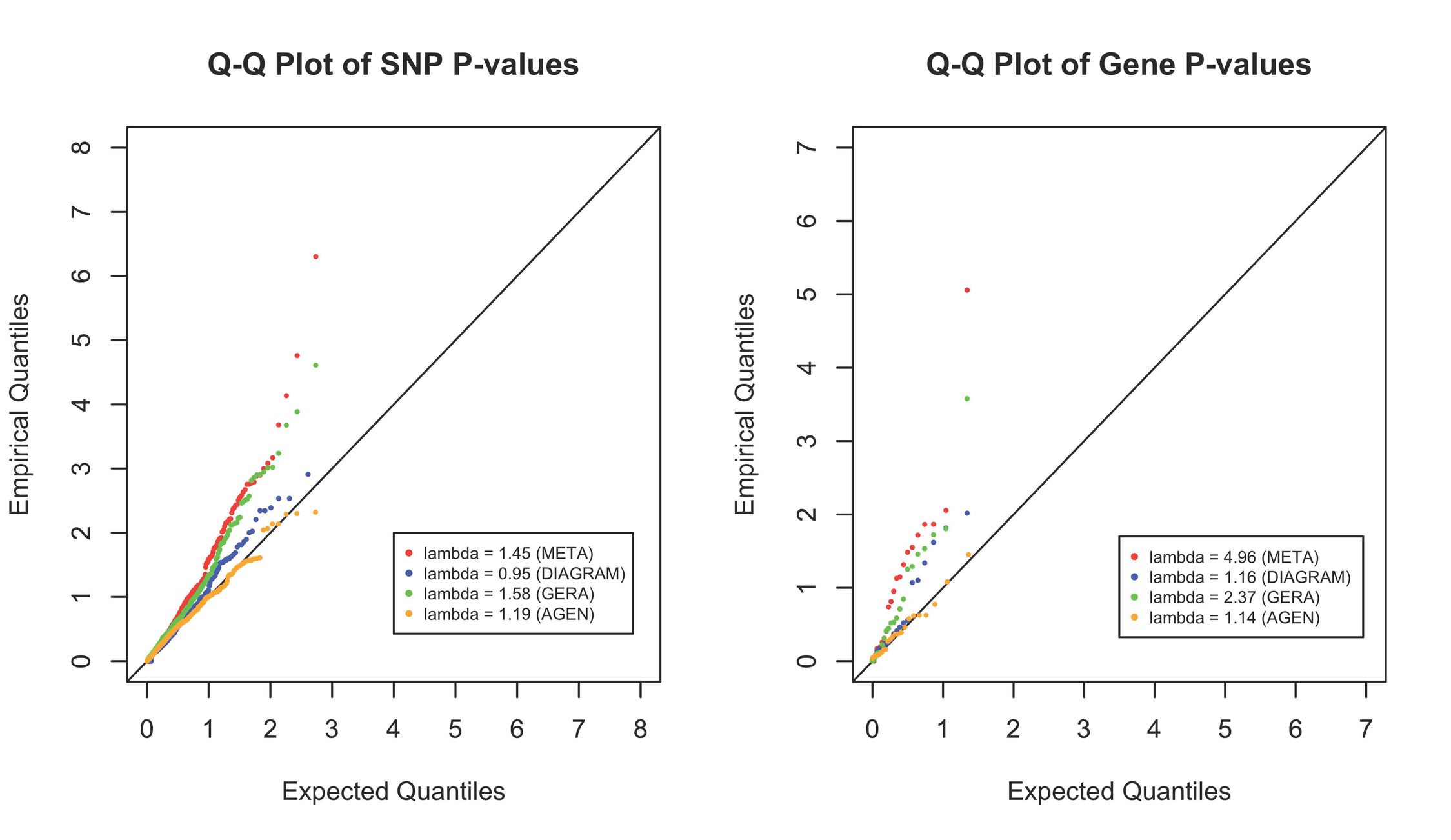

Supplement: S33 Fig — (TIF) [file pgen.1006122.s042.tif]

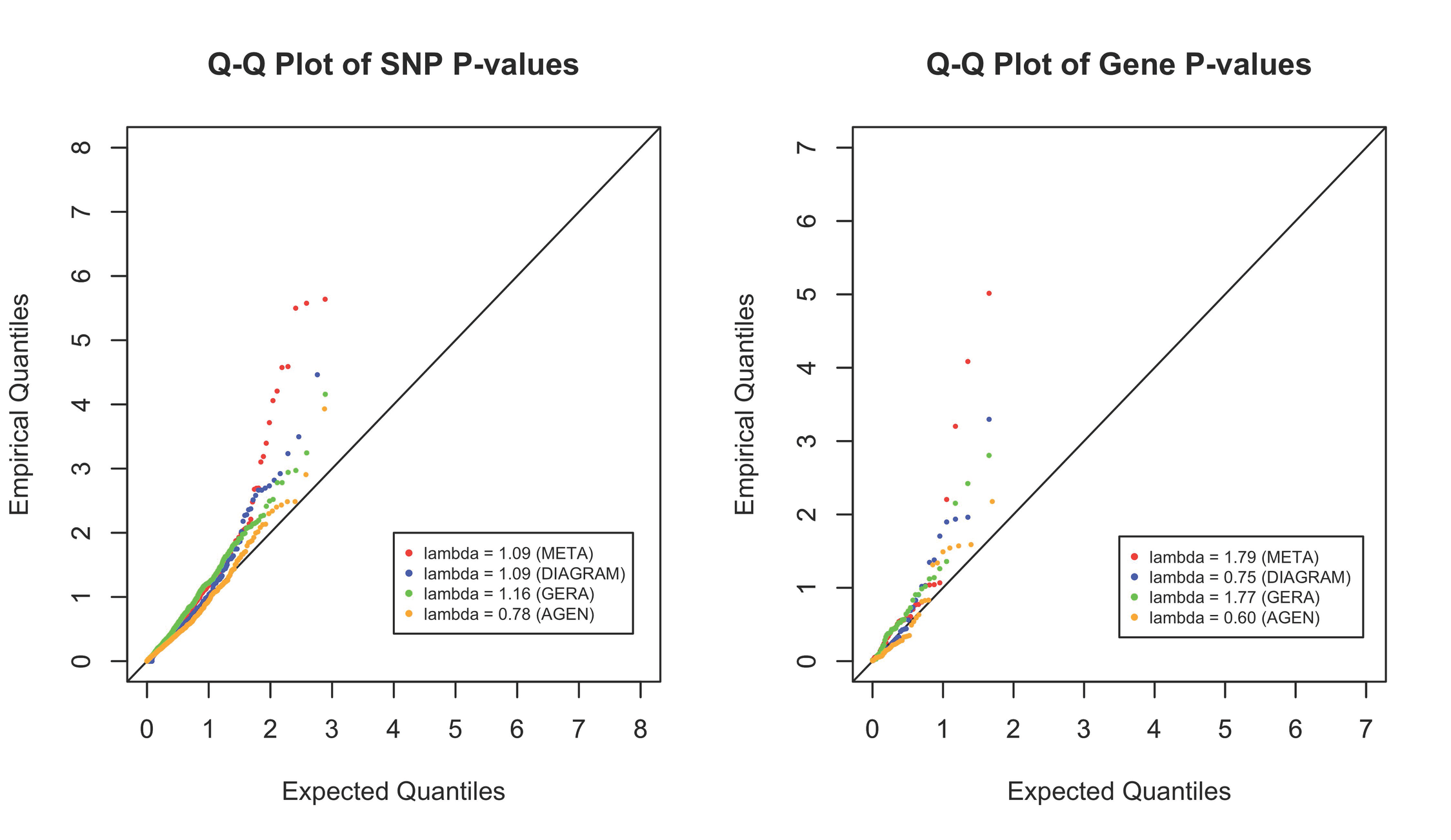

Supplement: S34 Fig — (TIF) [file pgen.1006122.s043.tif]

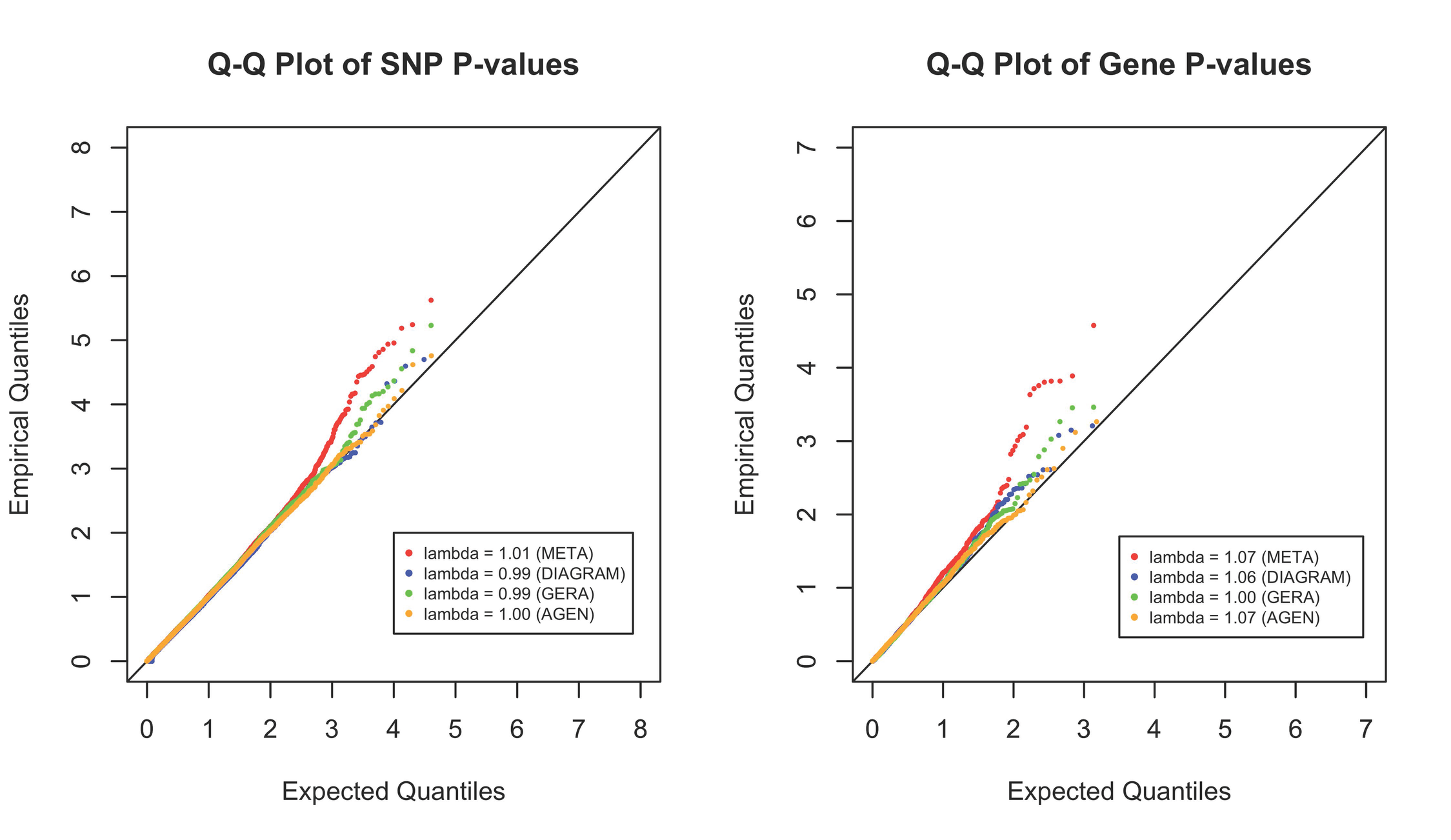

Supplement: S35 Fig — (TIF) [file pgen.1006122.s044.tif]

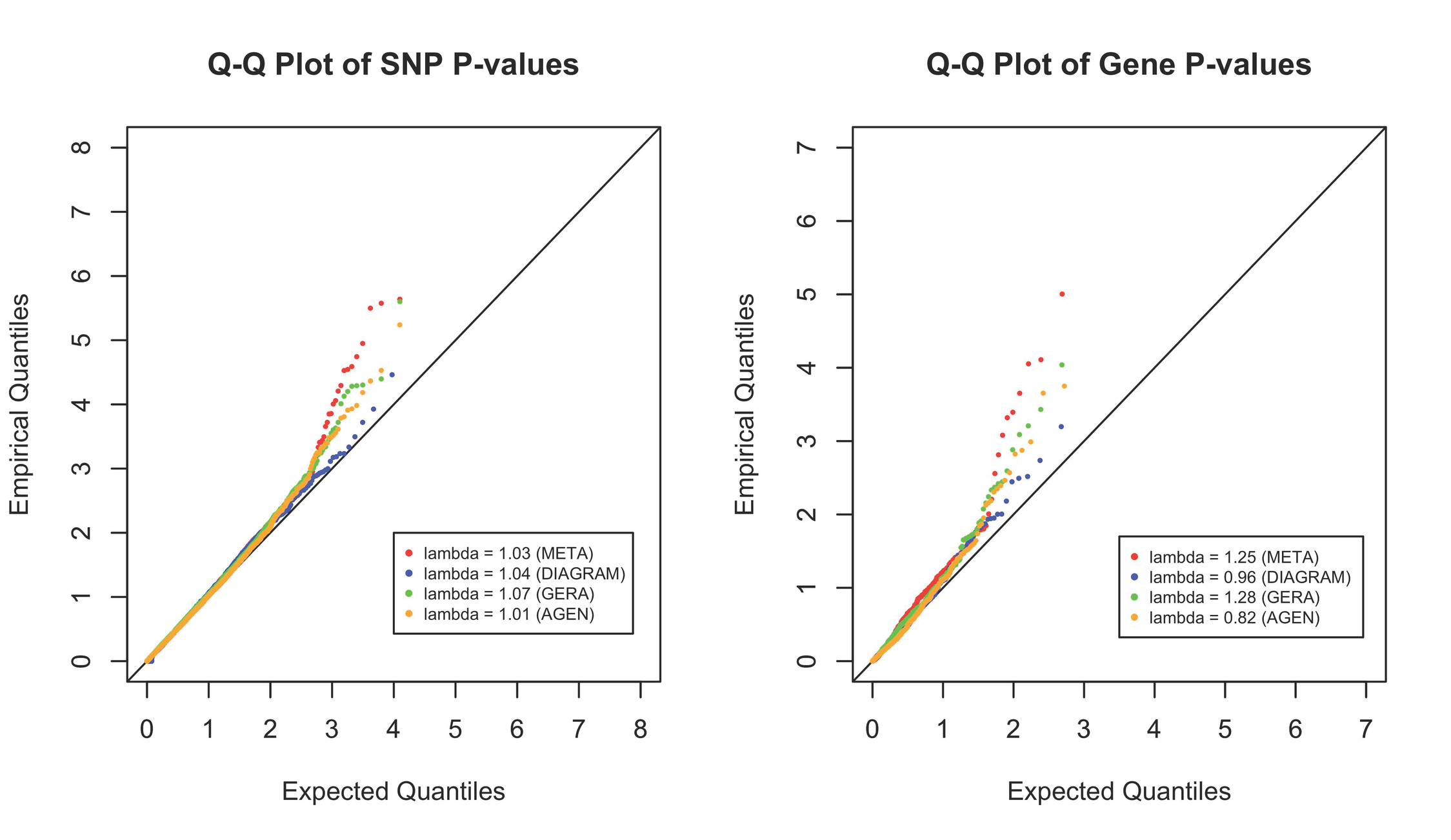

Supplement: S36 Fig — (TIF) [file pgen.1006122.s045.tif]

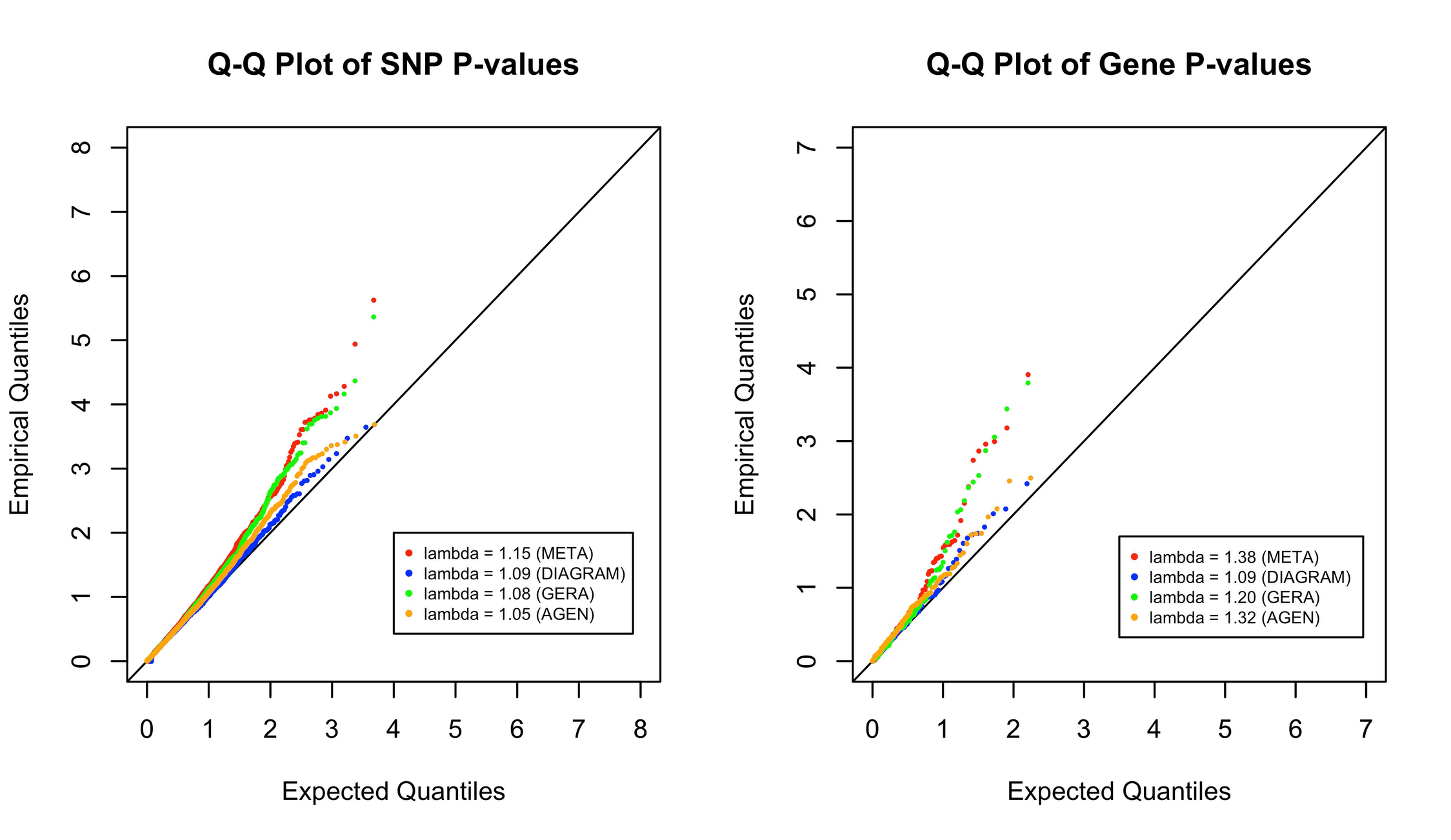

Supplement: S37 Fig — (TIF) [file pgen.1006122.s046.tif]

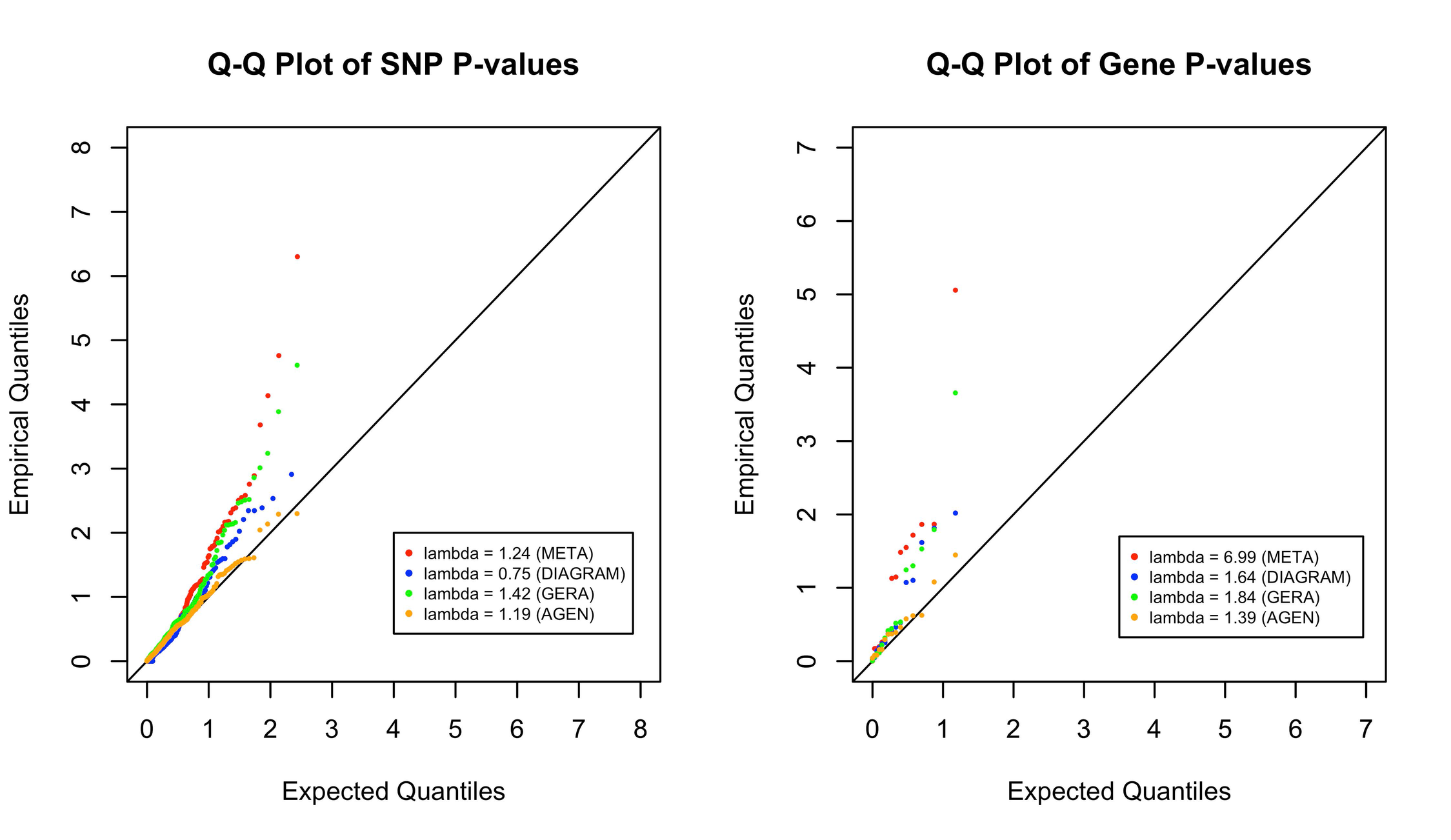

Supplement: S38 Fig — (TIF) [file pgen.1006122.s047.tif]

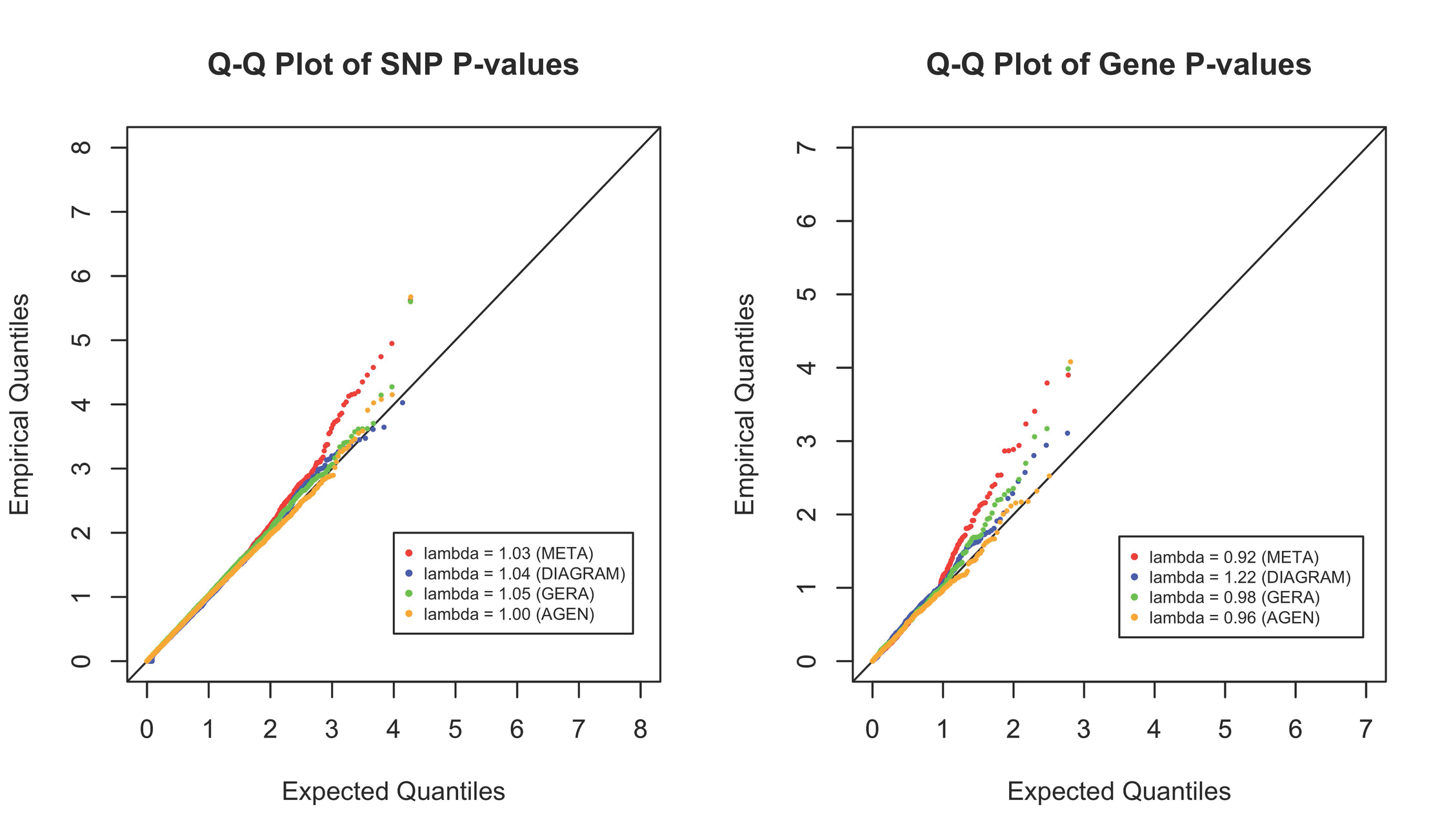

Supplement: S39 Fig — (TIF) [file pgen.1006122.s048.tif]

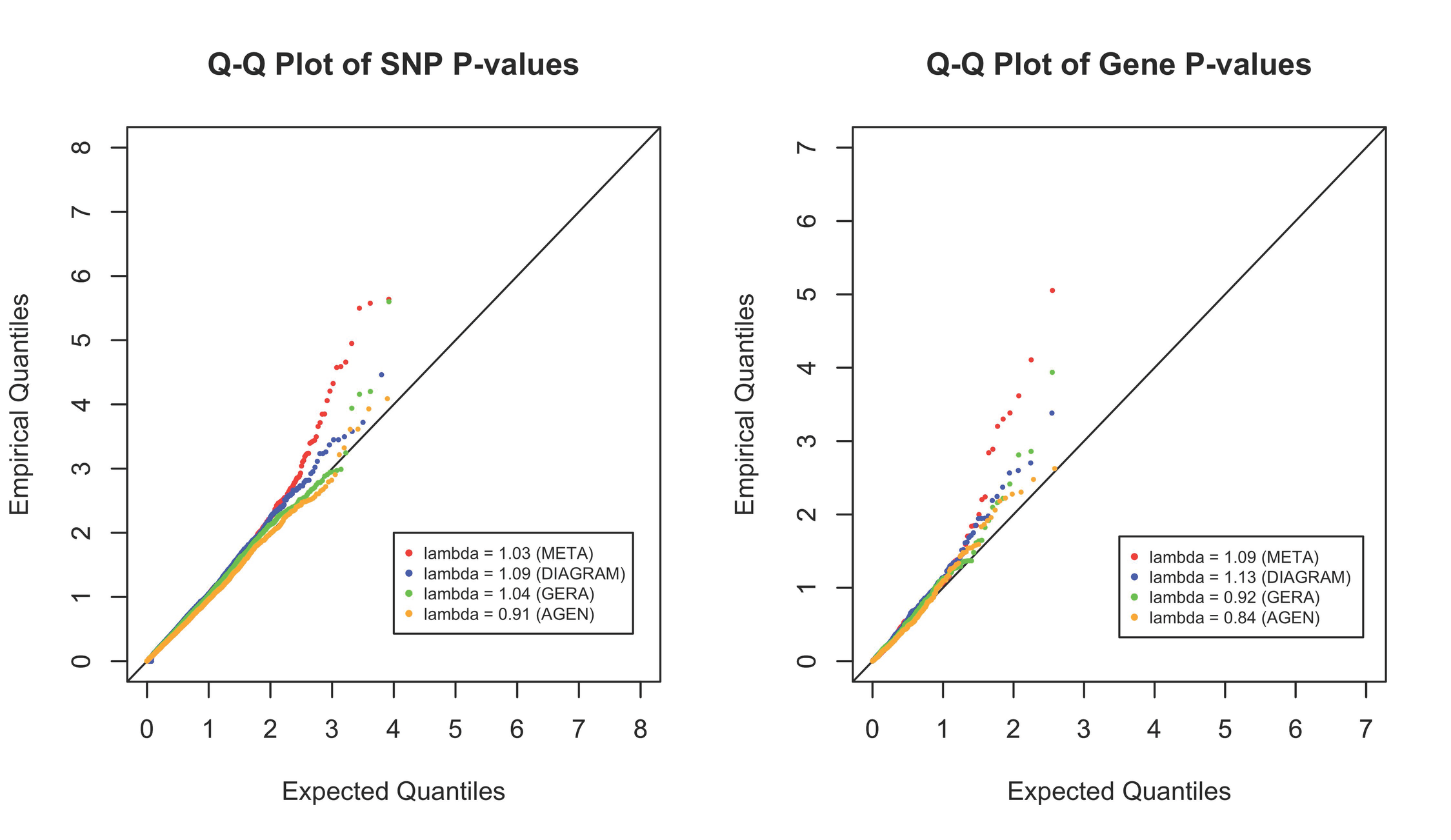

Supplement: S40 Fig — (TIF) [file pgen.1006122.s049.tif]

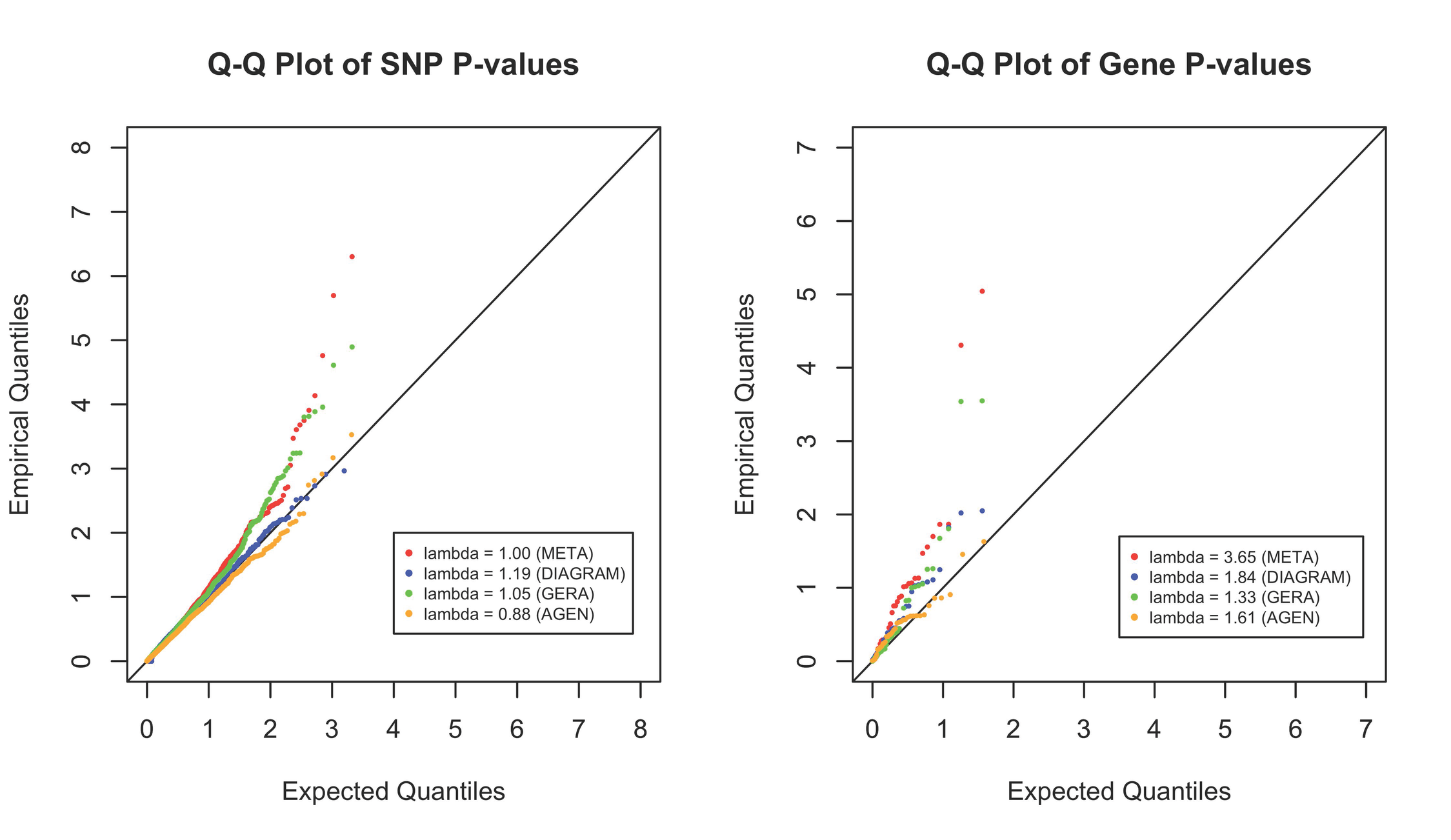

Supplement: S41 Fig — (TIF) [file pgen.1006122.s050.tif]

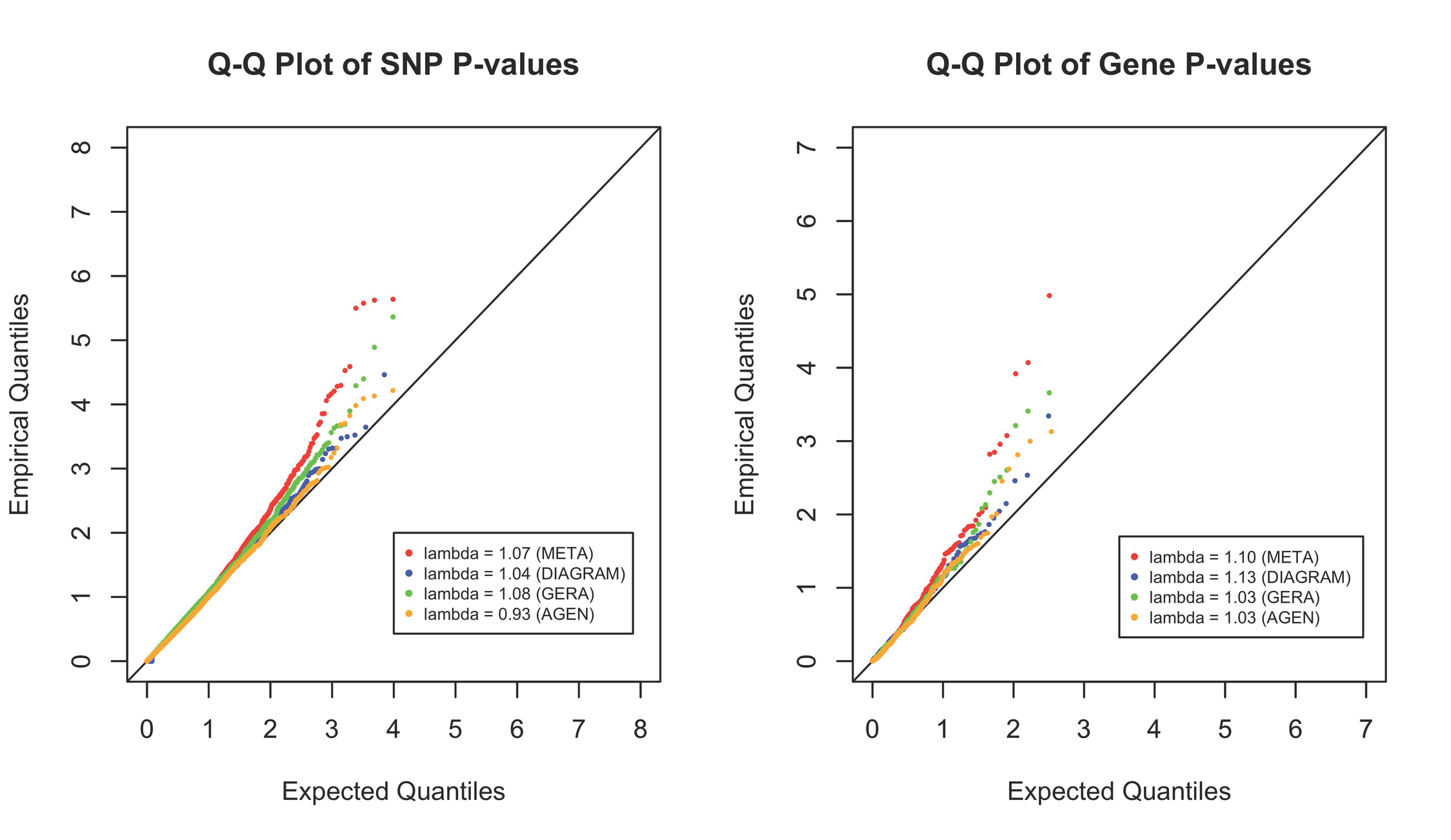

Supplement: S42 Fig — (TIF) [file pgen.1006122.s051.tif]

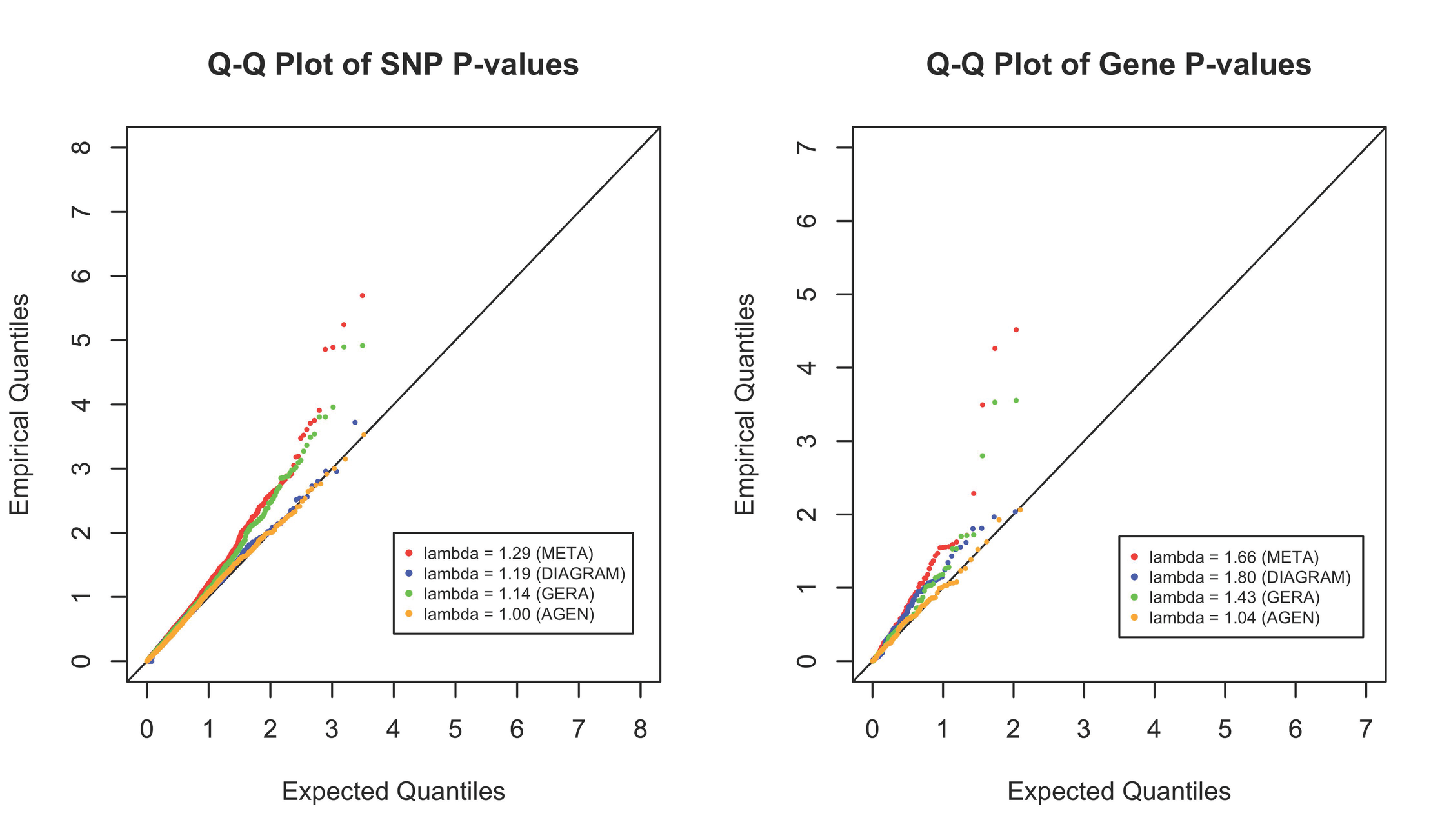

Supplement: S43 Fig — (TIF) [file pgen.1006122.s052.tif]

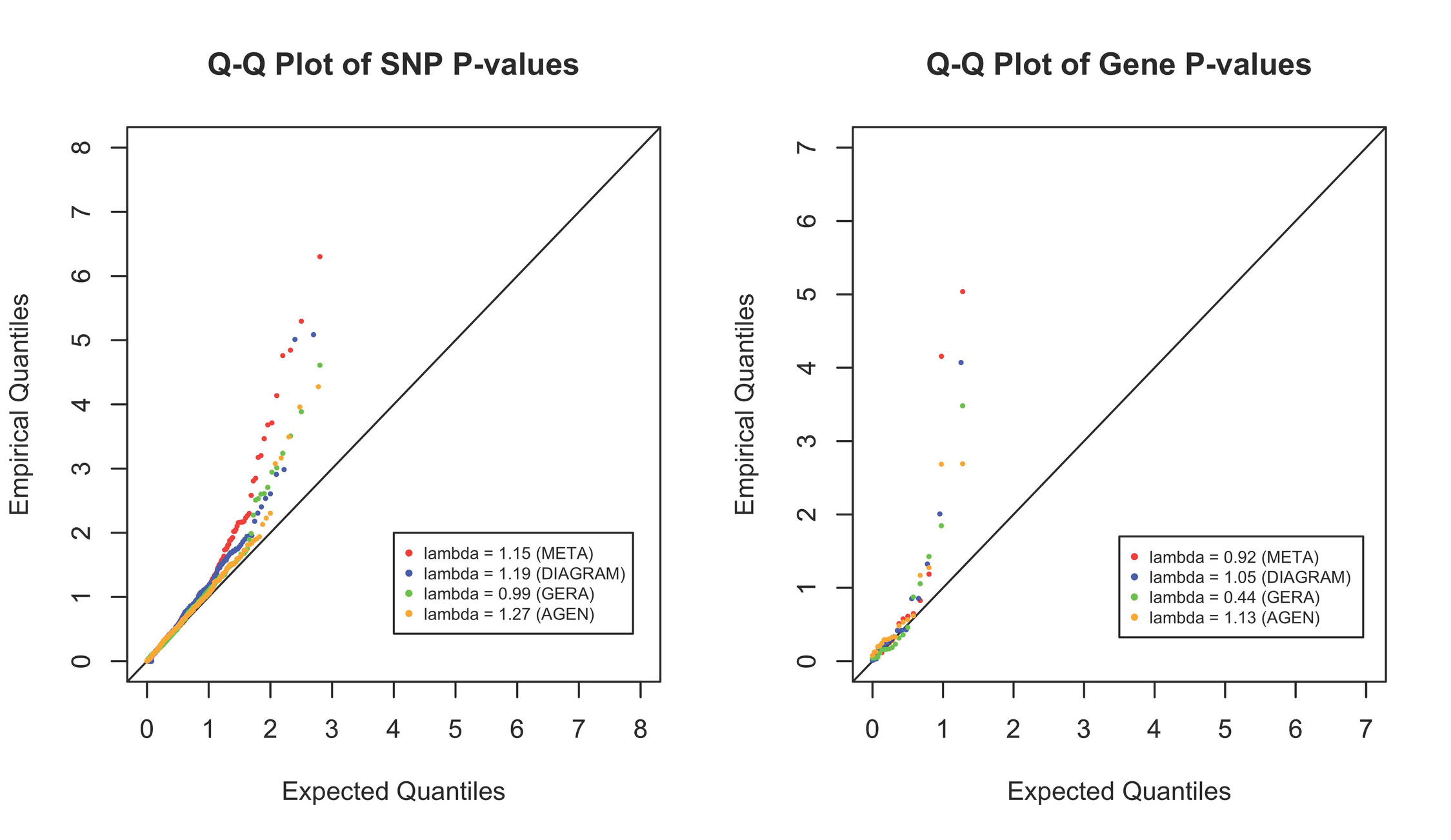

Supplement: S44 Fig — (TIF) [file pgen.1006122.s053.tif]

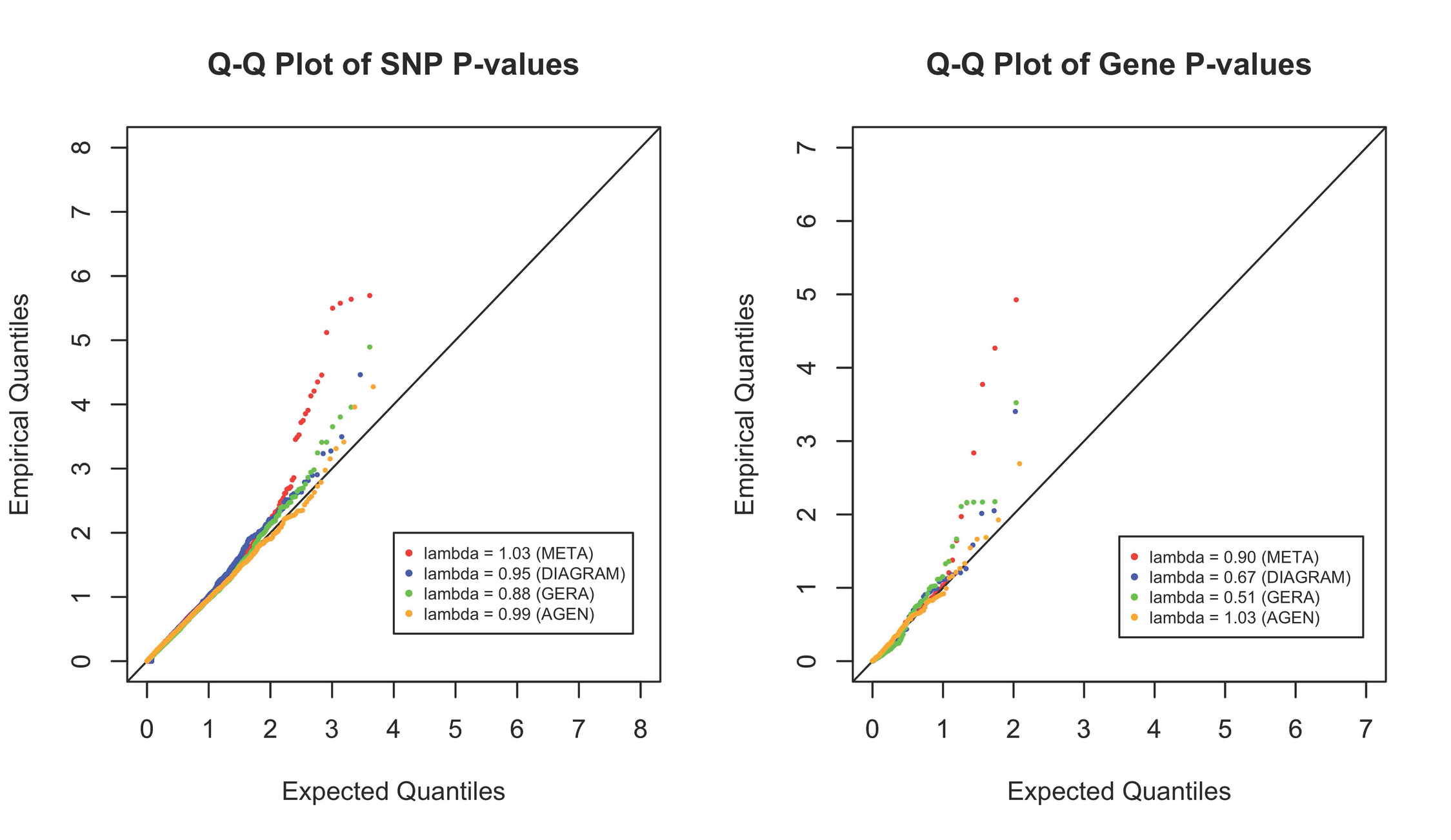

Supplement: S45 Fig — (TIF) [file pgen.1006122.s054.tif]

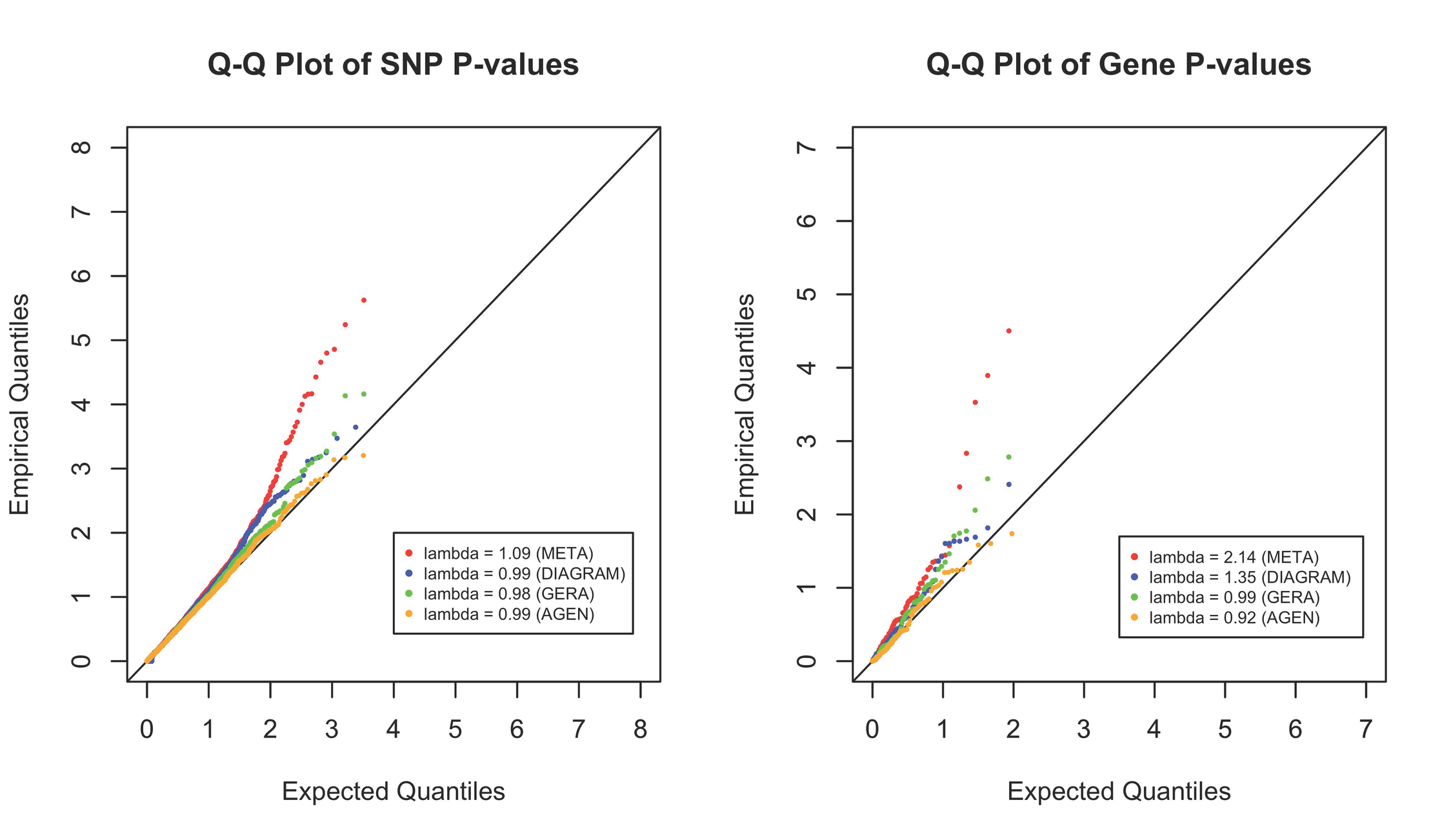

Supplement: S46 Fig — (TIF) [file pgen.1006122.s055.tif]

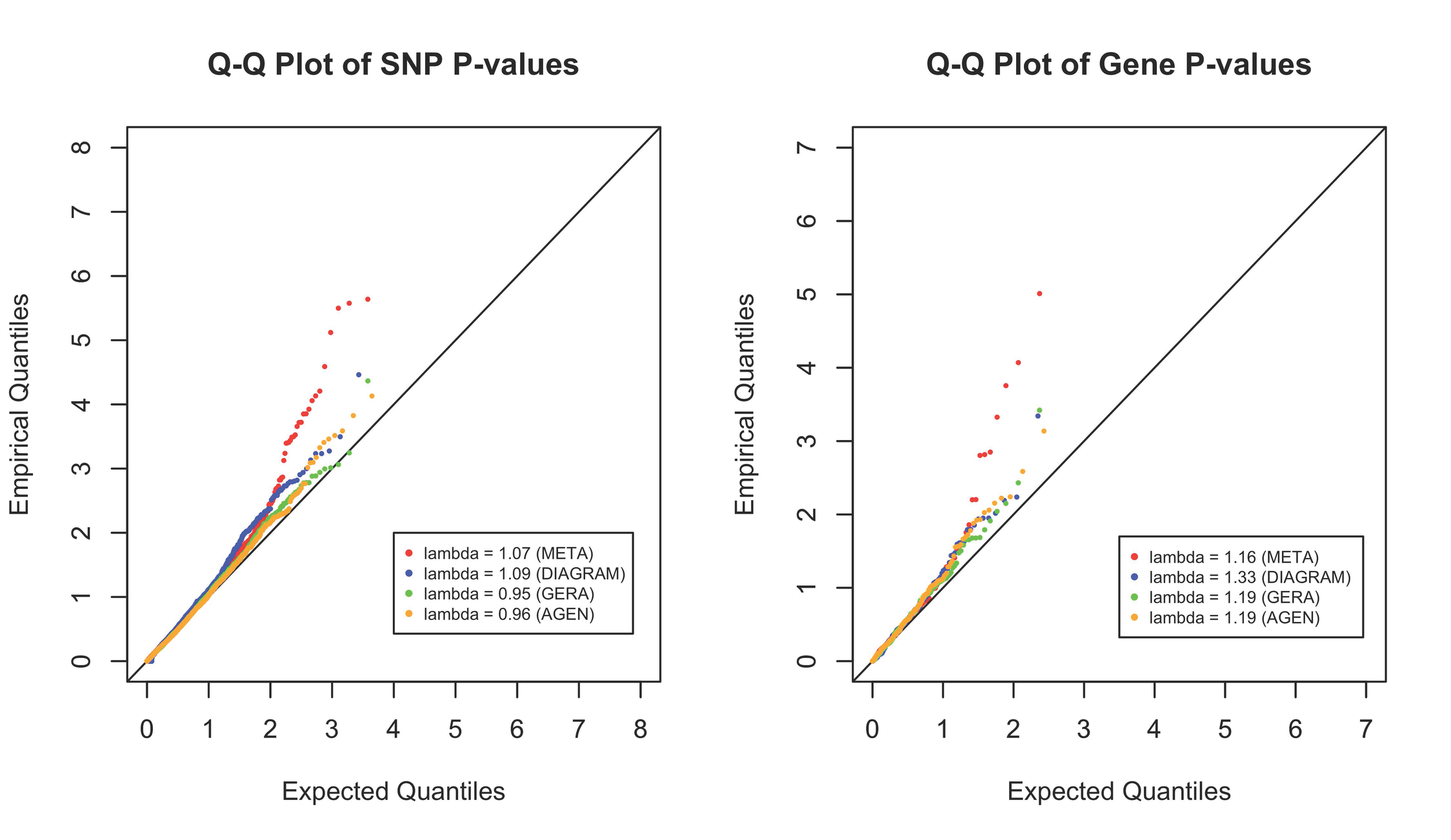

Supplement: S47 Fig — (TIF) [file pgen.1006122.s056.tif]

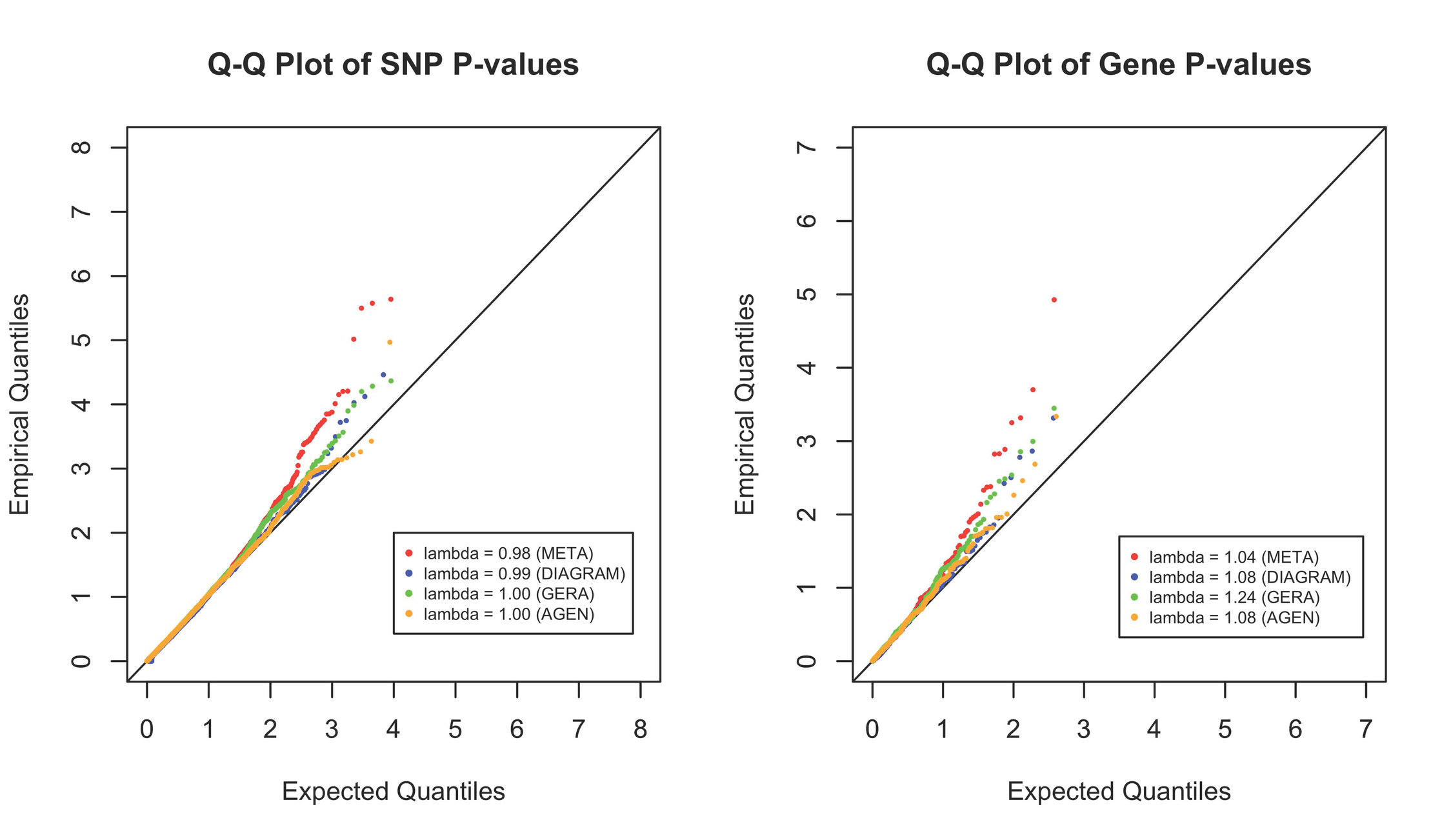

Supplement: S48 Fig — (TIF) [file pgen.1006122.s057.tif]

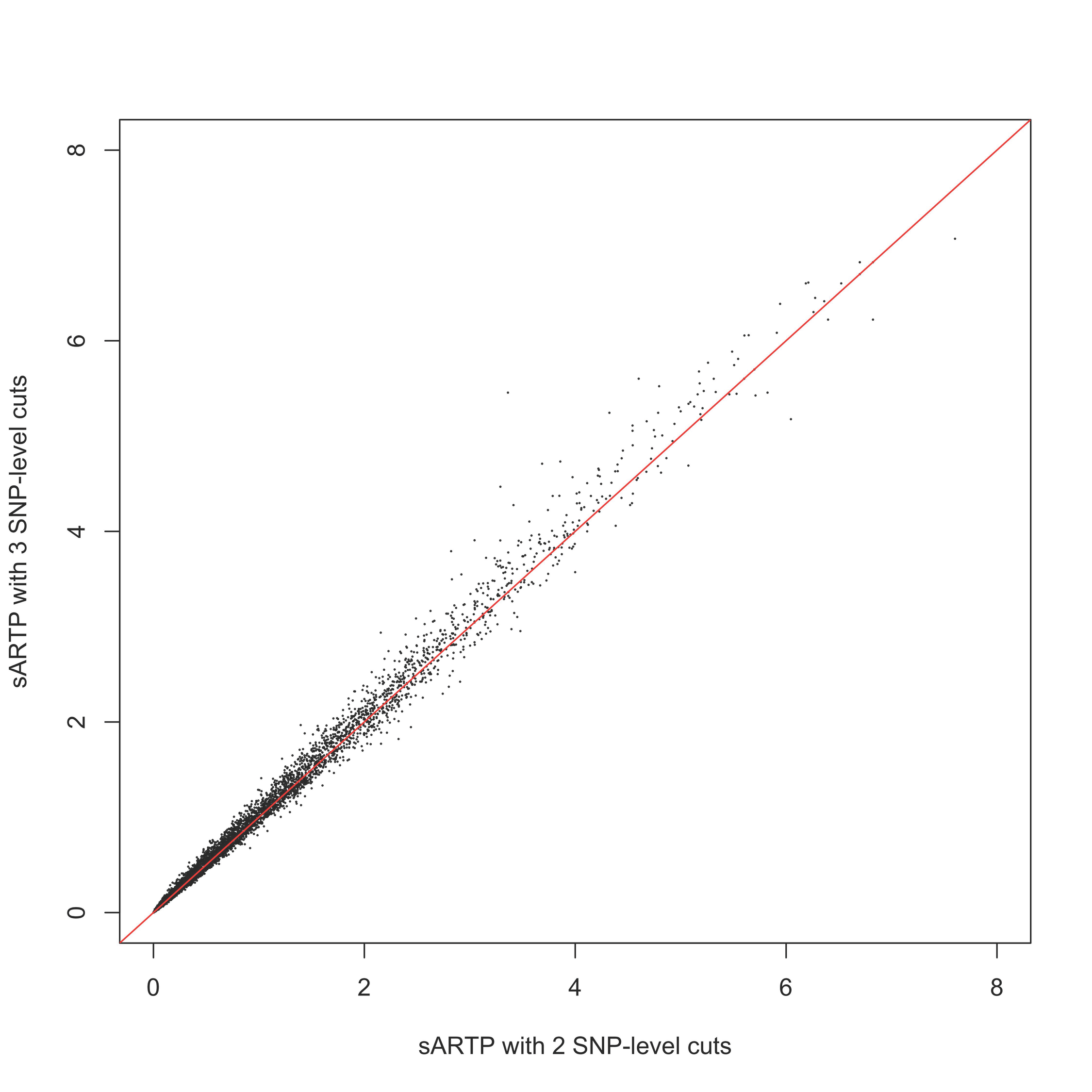

Supplement: S49 Fig — The p-values (in −log10 scale) of 4,713 pathways defined in MSigDB v5.0 were obtained with sARTP. (TIF) [file pgen.1006122.s058.tif]

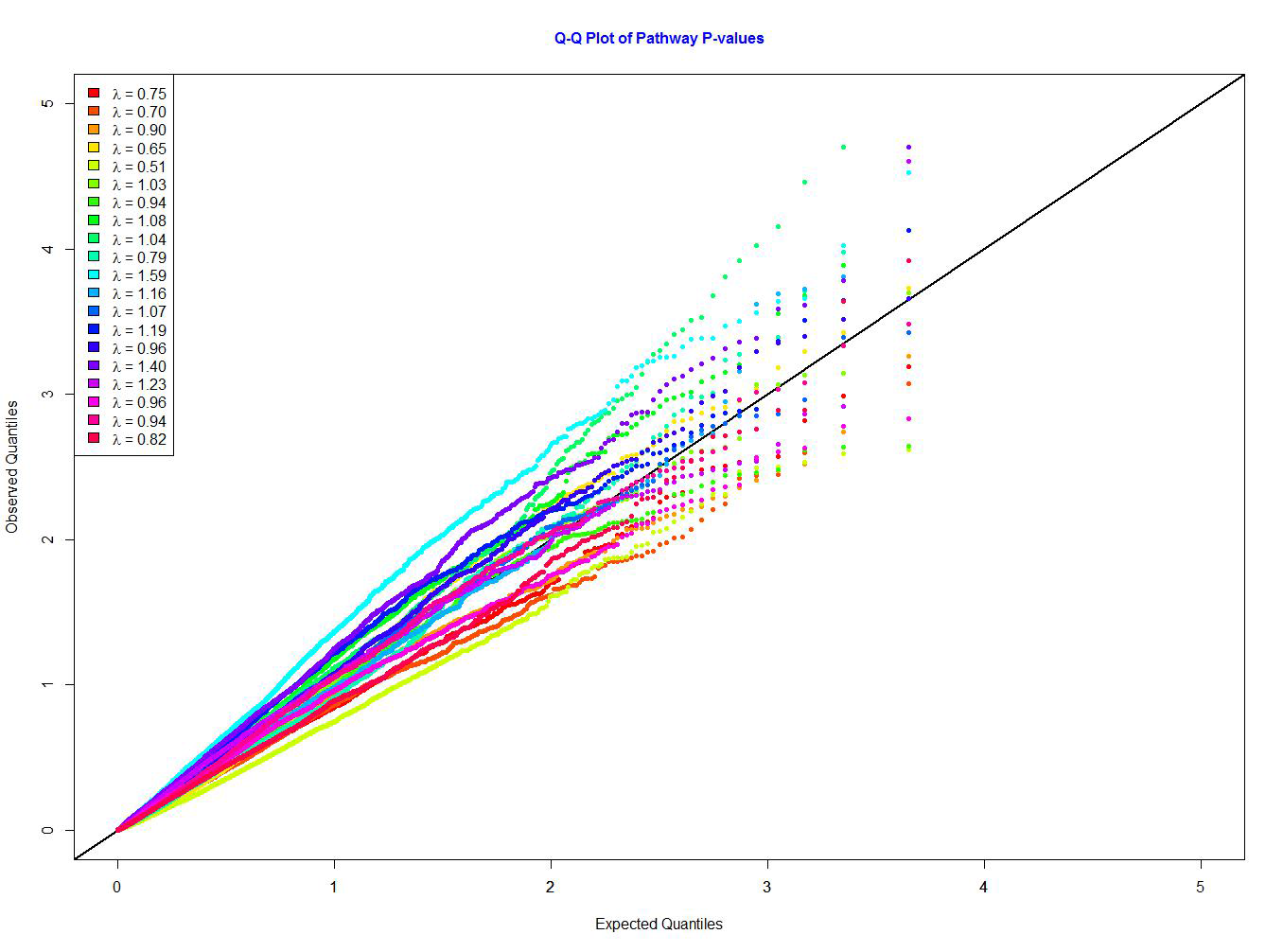

Supplement: S50 Fig — Based on each generated GWAS, 4,439 pathways (each with no more than 10,000 SNPs) defined in MSigDB v5.0 were analyzed with sARTP. λ is the genomic control inflation factor of the pathway p-values. (TIF) [file pgen.1006122.s059.tif]
